# Quantitative SUMO proteomics reveals the modulation of several PML nuclear body associated proteins and an anti-senescence function of UBC9

**DOI:** 10.1038/s41598-018-25150-z

**Published:** 2018-05-17

**Authors:** Francis P. McManus, Véronique Bourdeau, Mariana Acevedo, Stéphane Lopes-Paciencia, Lian Mignacca, Frédéric Lamoliatte, John W. Rojas Pino, Gerardo Ferbeyre, Pierre Thibault

**Affiliations:** 1 Institute of Research in Immunology and Cancer, Université de Montréal, Montréal, QC H3C 3J7 Canada; 2 Department of Biochemistry and Molecular Medicine, Université de Montréal, Montréal, QC H3C 3J7 Canada; 3 Department of Chemistry, Université de Montréal, Montréal, QC H3C 3J7 Canada

## Abstract

Several regulators of SUMOylation have been previously linked to senescence but most targets of this modification in senescent cells remain unidentified. Using a two-step purification of a modified SUMO3, we profiled the SUMO proteome of senescent cells in a site-specific manner. We identified 25 SUMO sites on 23 proteins that were significantly regulated during senescence. Of note, most of these proteins were PML nuclear body (PML-NB) associated, which correlates with the increased number and size of PML-NBs observed in senescent cells. Interestingly, the sole SUMO E2 enzyme, UBC9, was more SUMOylated during senescence on its Lys-49. Functional studies of a UBC9 mutant at Lys-49 showed a decreased association to PML-NBs and the loss of UBC9’s ability to delay senescence. We thus propose both pro- and anti-senescence functions of protein SUMOylation.

## Introduction

Many cellular mechanisms of defense have evolved to reduce the onset of tumors and potential cancer development. One such mechanism is cellular senescence where cells undergo cell cycle arrest in response to various stressors^1,2^. Multiple triggers for the onset of senescence have been documented. While replicative senescence is primarily caused in response to telomere shortening^3,4^, senescence can also be triggered early by a number of exogenous factors including DNA damage, elevated levels of reactive oxygen species (ROS), high cytokine signaling, and constitutively-active oncogenes (such as H-RAS-G12V)^5,6^. Dysregulation of HRAS has also been linked itself to aberrant levels of ROS due to mitochondrial dysfunction contributing to the senescent phenotype^7,8^. The node that connects all of these stressors to the same cellular fate remains elusive and has been the subject of much research. Although the biomarkers for senescence depend vastly on the basis of the stressor and cellular context, several markers have emerged such as the activation/upregulation of proteins from the DNA damage response pathway, increase in the number and the size of the promyelocytic leukemia (PML) nuclear bodies (PML-NBs), reduction in lamin B1 expression, depletion of linker histone as well as upregulation of p53 and some of its target genes^5,6,9–13^.

PML-NB formation is driven by the PML proteins. These nuclear structures are regulated by stress and allow for the sequestration of target proteins for their regulation and/or post-translational modification. PML-NBs have been implicated in senescence, DNA damage, apoptosis, defense against viral infection and elevated ROS levels^9,14^. They are also known to be hubs for the protein small ubiquitin-like modifier (SUMO). Indeed, several proteins including p53, DAXX, SP100, CBP, and ISG20 are known to accumulate in PML-NBs, and are also occasionally SUMOylated in this subnuclear compartment^15,16^. There is still debate as to what causes the nucleation of PML-NBs though earlier reports suggest that PML itself must be SUMOylated for body formation^17^. Since PML has a SUMO interacting motif (SIM) and at least three well documented SUMO sites, it is thought that the SUMOylated regions of a PML protein interacts with the SIM of the neighboring PML allowing for a polymerization effect, ultimately leading to the formation of the mature assemblies^18,19^. A recent paper by Lallemand-Breitenbach *et al*. refutes this hypothesis and supports a model where PML oligomerization into PML-NBs does not require SUMOylation, nor its SIM, but rather requires PML oxidation^14^. Upon PML-NB formation, UBC9 (the only SUMO conjugating enzyme) is recruited to the nuclear bodies where SUMOylation is activated toward PML^14^. Subsequently, proteins that contain a SIM will associate to the PML-NBs where they can also be SUMOylated. The SUMOylated partner proteins are ultimately sequestered in the PML-NBs via a SUMO-SIM interaction with PML.

Protein SUMOylation plays an important part in cellular functions and has been linked to changes in DNA repair, intracellular trafficking, cell signaling and stress responses^20–24^. Protein SUMOylation is a post-translational modification (PTM) that is targeted to the ε-atom of certain Lys residues resulting in branched protein formation. The SUMO pathway draws a high degree of parallel with the ubiquitylation network. Both mechanisms necessitate a machinery with three enzymatic activities: E1 (activating), E2 (conjugating) and E3 (ligating)^25^. Recent advances in proteomics based approaches that allow to identify SUMO sites in large scale studies has granted much insight into substrate SUMOylation since the motif/region of the protein being modified can be identified. Vertegaal *et al*. used a Lys-deficient SUMO3 protein with a N-terminal His-tag to achieve SUMO site identification^26^. Though this method has yielded roughly 4000 SUMO sites, this avenue is not optimal for biological assays since polySUMOylation may play a key role in the cell. Other groups have used the already available α-GlyGly antibody to recognize SUMO3 sites, while our group has developed a new antibody specific for our modified SUMO3 remnant for site identification^26–29^.

The aim of this study is to characterize the role of SUMOylation in H-RAS-G12V-mediated cellular senescence. The potential role of protein SUMOylation in cellular senescence is relevant considering the crosstalk between this modification and ubiquitylation. Protein degradation by the proteasome, presumably due to ubiquitylation, is linked to the senescent phenotype^30^. Moreover, SUMOylation likely plays a role in senescence considering that increased levels of ROS are detected during senescence and have the potential to activate PML-NB formation and promote substrate SUMOylation and sequestration^14^.

Our SUMO3 proteome of H-RAS-G12V-induced senescence reports 25 SUMO sites that are significantly regulated in senescence. Many of the targeted proteins are associated to PML-NBs. Moreover, the E2 SUMO ligase UBC9 was found to be more SUMOylated during senescence, suggesting an unsuspected role of UBC9 SUMOylation that serves an anti-senescence function.

## Results

### Profiling changes in the SUMO proteome of senescent cells

**Figure 1:**
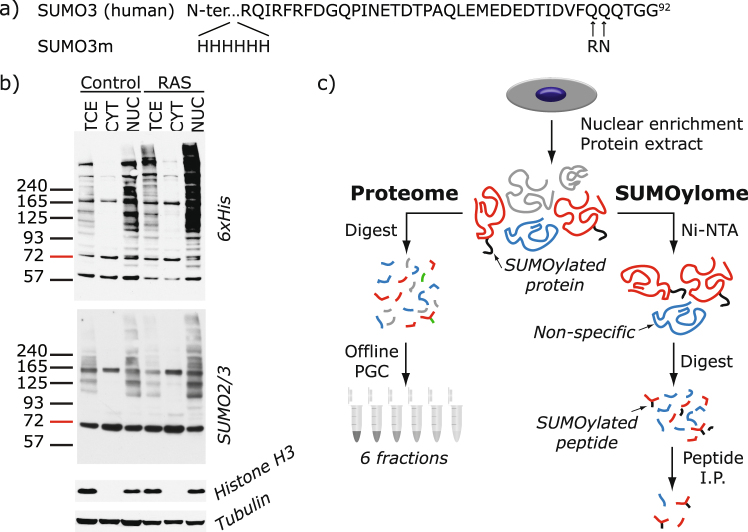
Method for SUMO Site Identification. (**a**) Amino acid sequence of human SUMO3 and the modified SUMO3 (SUMO3m) used in this work. A 6xHis tag was introduced at the protein N-terminus for Ni-NTA purification. Q87R and Q88N alterations were introduced for trypsin cleavage and peptide identification purposes, respectively. (**b**) Western blots for 6xHis, SUMO2/3, histone H3 or tubulin of total cell extract (TCE), cytoplasmic fraction (CYT) and nuclear enriched fraction (NUC) from U2OS cells expressing SUMO3m and an empty vector (Control) or H-RAS-G12V (RAS). (**c**) Workflow adopted for SUMO3 site identification and proteome quantification. U2OS cells expressing SUMO3m were transduced with either an empty or RAS expressing vector. Cells were collected 10 days after transduction and fractionated under hypotonic conditions. For SUMO site identification, the nuclear enriched fractions were subjected to Ni-NTA purification, followed by trypsin digestion and finally subjected to immunopeptide enrichment with an antibody recognizing the NQTGG SUMO remnant left on the peptide backbone. For proteome analysis, the nuclear proteins were digested with trypsin, desalted and fractionated by PGC. The resulting peptides were analyzed on a Q-exactive plus mass spectrometer and data analyzed using MaxQuant.

To determine changes in protein SUMOylation and identify modification sites in the proteome of senescent cells, we used a recently developed quantitative proteomic approach whereby SUMO proteins are first enriched on an Ni-NTA column prior to tryptic digestion, immunoaffinity purification of SUMO remnant peptides, and analysis by mass spectrometry (MS) (Fig. 1a). We chose to employ our previously developed methodology for SUMO site identification for this study since the modified SUMO3 (SUMO3m) construct has been studied extensively and has been shown to conjugate like the native SUMO3 protein and deconjugate readily by the SENP proteins^31,32^. Specific enrichment of SUMO peptides was applied to nuclear enriched extracts of growing or senescent U2OS cells stably expressing our SUMO3m (Fig. 1a for sequence) containing a N-terminus His tag and a C-terminus Q87R-Q88N modification that upon tryptic digestion releases a five amino acid SUMO remnant recognized by a custom antibody^28^.

Senescence was induced in human osteosarcoma U2OS cells using the oncogene H-RAS-G12V that promotes a senescence growth arrest in these tumor cells, independently of the p16 and ARF tumor suppressors^33^. We therefore introduced a control vector or a vector expressing oncogenic H-RAS-G12V (referred to as RAS) in U2OS cells modified to express the SUMO3m. We studied the resulting senescent phenotype once well established at 10 days following RAS transduction. The mRNA levels of several key markers of the senescence phenotype were validated by quantitative PCR (qPCR) to ensure that RAS-induced senescence in the U2OS cells expressing the SUMO3m construct followed a canonical senescence reprograming (Supplementary Fig. S1). These *bona fide* markers include the increase in the levels of the p53 target genes CDKN1A/p21 and GADD45A, decrease in lamin B1 expression, reduction in RB/E2F targets and the cell proliferation biomarker KI67, induction of expression of an autophagy gene and the dramatic increase in interleukin 1-beta and 8^34^. To ascertain the effect of RAS-mediated senescence on the global SUMO proteome and to identify subcellular location of protein SUMOylation, cells were fractionated into the cytoplasmic and nuclear-enriched fractions prior to western blotting (Fig. 1b). We noticed that >90% of the SUMO2/3 signal was observed in the nuclear fraction. Accordingly, we opted to fractionate samples and use the nuclear-enriched fraction for SUMO site identification and quantification. Interestingly, the global SUMOylation pattern was increased upon senescence and the greatest gain in SUMO signal occurs at high molecular weight, suggesting a role for polySUMOylation (Fig. 1b).

Our tandem purification protocol using nickel-nitrilotriacetic acid (Ni-NTA) and immunoprecipitation (IP) against the SUMO3m remnant (Fig. 1c) yields peptide samples containing more than 30% SUMO peptides. Our methodology allowed for the identification of 244 SUMO sites (localization probability >0.75) on 168 proteins (Supplementary Table S1 and Supplementary Fig. S2a). Changes in the proteome were also investigated to validate that RAS-mediated senescence phenotype was observed in all our senescent samples. Accordingly, we fractionated U2OS protein extracts into 6 fractions using basic porous graphite chromatography (PGC) separation to extend the comprehensiveness of proteome analysis. Quantitative proteomic analyses enabled the profiling of 1738 proteins, of which 32 proteins were identified as SUMO substrates. These analyses also revealed that 65 proteins were significantly regulated during senescence (5% FDR) (Supplementary Fig. S3 and Supplementary Table S2), but only 2 of these were also SUMOylated.

### Senescence Affects the SUMOylation of PML-Associated Proteins

**Figure 2:**
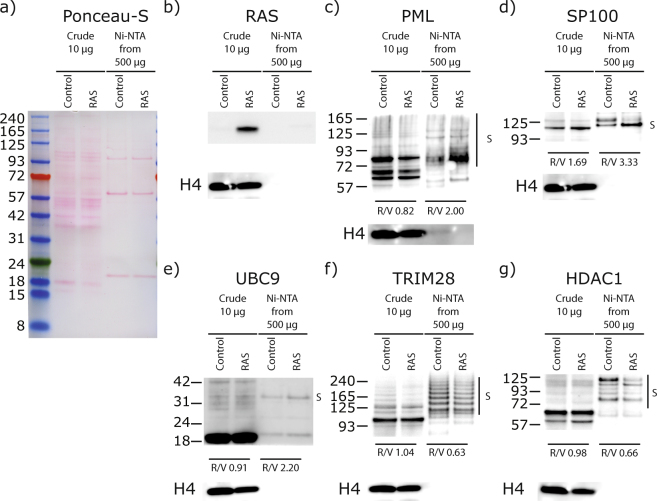
Western blot validation of SUMO targets regulated in senescence. (**a**) Ponceau-S staining of 10 μg of nuclear enriched proteins (Crude) transferred on nitrocellulose showing the constant loading between U2OS expressing SUMO3m plus a control vector (Control) or a vector expressing H-RAS-G12V (RAS) to induce senescence. Extracts from both conditions were subjected to Ni-NTA purification to purify proteins SUMOylated with SUMO3m and used for blotting in panels c through g. (**b**) Anti-RAS blotting depicting the increased levels of steady state RAS in the senescent cells (crude extracts as in a). (**c**,**d**) Immunoblots showing an increase in total protein and in SUMOylated PML (**c**) and SP100 (**d**) in the senescent samples (cells as in **a**). (**e**) Anti-UBC9 blot showing an increased SUMOylated UBC9 in the senescent samples (cells as in a). (**f**–**g**) Immunoblots showing a decrease in SUMOylated TRIM28 (**f**) and in SUMOylated HDAC1 (**g**) in the senescent samples (cells as in **a**). Histone H4 serves as a loading control for crude extracts in (**b**–**g**).

Out of the 244 SUMO sites identified (Supplementary Data S1 and Supplementary Table S1), 25 were statistically regulated by RAS-mediated cellular senescence based on the analysis of five biological replicates: 13 sites were increased upon senescence while 12 were decreased (Table 1). These 25 regulated SUMO sites are located on 23 proteins, 8 of which are directly associated with PML-NBs, representing roughly a third of all regulated targets (gene names shown in bold in Table 1 represent proteins known to be associated with PML-NBs)^35^. We validated five of the proteins identified with SUMOylation changes by western blot analysis (Fig. 2). The western blot results corroborated the MS data, where we observed an increase in SUMOylation for PML, SP100 and UBC9 and a decrease in SUMOylation of TRIM28 and HDAC1 in senescent cells. Of note, we did observe a second SUMO band on SP100 in the Ni-NTA enriched samples for the control sample that was not found in the RAS treatment (Fig. 2d). Though we do not know the nature of this band other than it is SUMOylated, we cannot rule out the possibility that SP100 is polySUMOylated (SUMOylation on the SUMO protein that is conjugated at Lys-297 on SP100) or that it is SUMOylated on both Lys-297 and Lys-306 under basal conditions (SUMO sites reported in phosphosite plus) and that this double or polySUMOylation is lost during senescence.

**Table 1:** List of SUMO Sites Regulated by Senescence.

Protein	Gene Name	SUMO site	FC^a^	Unique^b^	Known PTM^c^
78 kDa glucose-regulated protein	HSPA5	352	29.58	x	Sm, Ac, Ub
Actin, cytoplasmic 1	ACTB	113	16.59	x	Sm, Ac, Ub
Ribosome biogenesis protein BRX1 homolog	BRIX1	322	15.58	x	Sm, Ub
NGFI-A-binding protein 1	NAB1	480	8.86		Sm
Promyelocytic leukemia protein	**PML**	490	8.53		Sm, Ac
Class E basic helix-loop-helix protein 40	BHLHE40	279	8.00		Sm
Exosome component 10	EXOSC10	583	6.74	x	Sm
Unconventional myosin-Ib	MYO1B	287	5.65		Sm, Ub
Small ubiquitin-related modifier 3	**SUMO3**	41	3.72		Sm, Ac, Ub
Small ubiquitin-related modifier 2	**SUMO2**	42	3.72		Sm, Ac, Ub
Nuclear autoantigen Sp-100	**SP100**	297	3.57		Sm
SUMO-conjugating enzyme UBC9	**UBE2I/UBC9**	49	3.36		Sm, Ub
MORC family CW-type zinc finger protein 3	**MORC3**	740	3.13		Sm
Zinc finger MYM-type protein 4	ZMYM4	250	0.74		Sm
Scaffold attachment factor B2	SAFB2	230	0.74		
Putative oxidoreductase GLYR1	**GLYR1**	176	0.57		
Transcription intermediary factor 1-beta	TRIM28	779	0.41		Sm, Ac, Ub
Transcription intermediary factor 1-beta	TRIM28	377	0.36		Sm, Ac, Ub
Histone deacetylase 1	**HDAC1**	476	0.31		Sm
Symplekin	SYMPK	483	0.24		Sm
Zinc finger protein 646	ZNF646	1168	0.19		Sm
Cleavage stimulation factor subunit 2	CSTF2	189	0.17		Ub
DNA repair protein complementing XP-C cells	XPC	81	0.16		Sm
B-cell lymphoma/leukemia 11A	BCL11A	799	0.15		
DNA repair protein complementing XP-C cells	XPC	89	0.13		

^a^FC: fold-change of SUMO site abundance in senescent cells divided by their abundance in controls cells, P-value < 0.05.

^b^Unique SUMO sites are defined as SUMO sites only observed in the senescent samples. FC are estimated using imputations of 1.8 standard deviations below the median for samples lacking a peptide abundance.

^c^Known PTM reported at this site based on “PhosphoSite Plus” accessed on February 8, 2018.

Sm: SUMOylation; Ac: Acetylation; Ub: Ubiquitylation.

Gene names in bold correspond to proteins known to be associated with PML-NBs.

**Figure 3:**
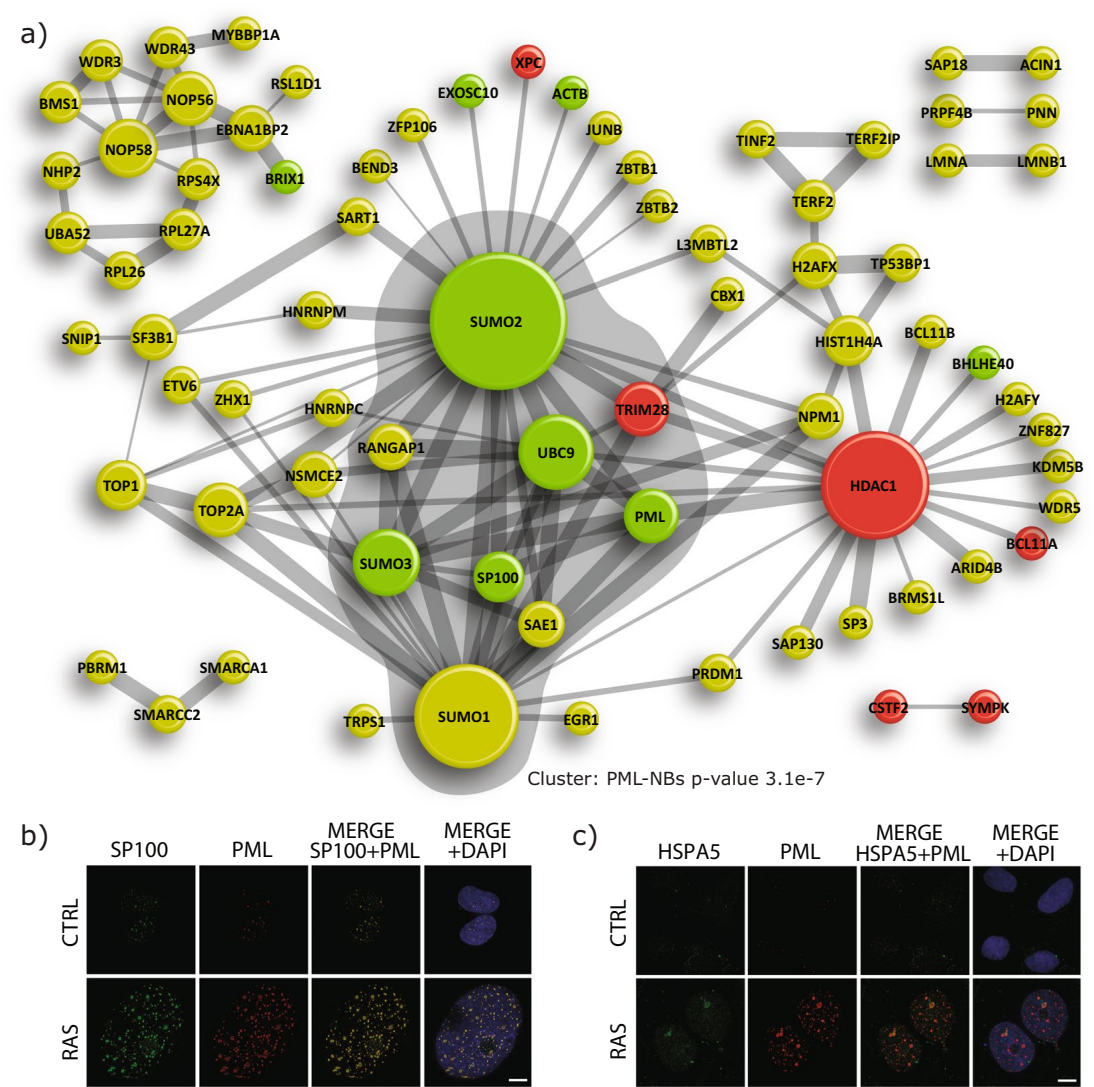
SUMOylated Protein Network. (**a**) STRING network of all SUMOylated proteins with a high interaction confidence (0.7 or greater). Proteins whose SUMOylation sites were statistically increased upon RAS-mediated senescence are represented in green and those whose SUMOylation sites were decreased are shown in red (p-value < 0.05). The size of the nodes correlate to the number of interactors while the size of the edge depicts the confidence of the interactions. Highlighted in grey is the highest scoring cluster extracted using MCODE. (**b**) Confocal immunofluorescence with anti-SP100 and anti-PML antibodies to show colocalization and induction of PML-NBs upon senescence in U2OS cells expressing SUMO3m plus a control vector (control) or a vector expressing RAS (scale bar, 10 μm). (**c**) Confocal immunofluorescence with anti-HSPA5 and anti-PML antibodies to probe for colocalization upon senescence of cells as in (**b**) (scale bar, 10 μm).

Motif analysis around all the SUMO3 sites identified in U2OS was virtually identical to those reported in the literature by both our group and others for HeLa and HEK293 cells, where SUMO sites reside primarily in the consensus motif ψK*x*E (where ψ is a hydrophobic residue and *x* any amino acid) (Supplementary Fig. S2b)^26,28,29^. Motif analysis comparing the senescence regulated SUMO sites to those identified in the global SUMO proteome analyses revealed no enrichment of amino acid or motif around the site of SUMOylation (Supplementary Fig. S2c). To gain further insights into the role of SUMOylation in senescence, we performed a network analysis of the identified SUMO proteome to determine the interconnectivity of proteins and the enrichment of particular pathways (Fig. 3a). MCODE was used to extract the tightest subnetwork from the primary network. The tightest subnetwork comprised SUMO1, SUMO2, SUMO3, SAE1, UBC9, PML, SP100 and TRIM28, and was found to be associated with PML-NBs. More interestingly, this subnetwork harbored 6 of the 23 proteins whose SUMOylation was regulated by senescence. This observation suggests that changes in SUMOylation occurring during senescence could be associated with PML-NBs or contribute to the target’s localization to nuclear bodies. This is consistent with the increase in the size (~2.5 time bigger) and number (~2.5 times more) of the PML-NBs observed upon RAS-mediated senescence (Fig. 3b)^36^. Interestingly, one of the strongly regulated SUMO site we identified was found on HSPA5 (also known as GRP78), a protein that usually resides in the endoplasmic reticulum. We performed immunofluorescence experiments on HSPA5 and found that HSPA5 is partly located in the PML-NBs only in RAS senescent cells (Fig. 3c). This unexpected relocalization of HSPA5 highlights the possibility that many other proteins from the list of regulated SUMO sites maybe residents of the PML-NBs under specific conditions.

### Important role of UBC9’s Lys-49

**Figure 4:**
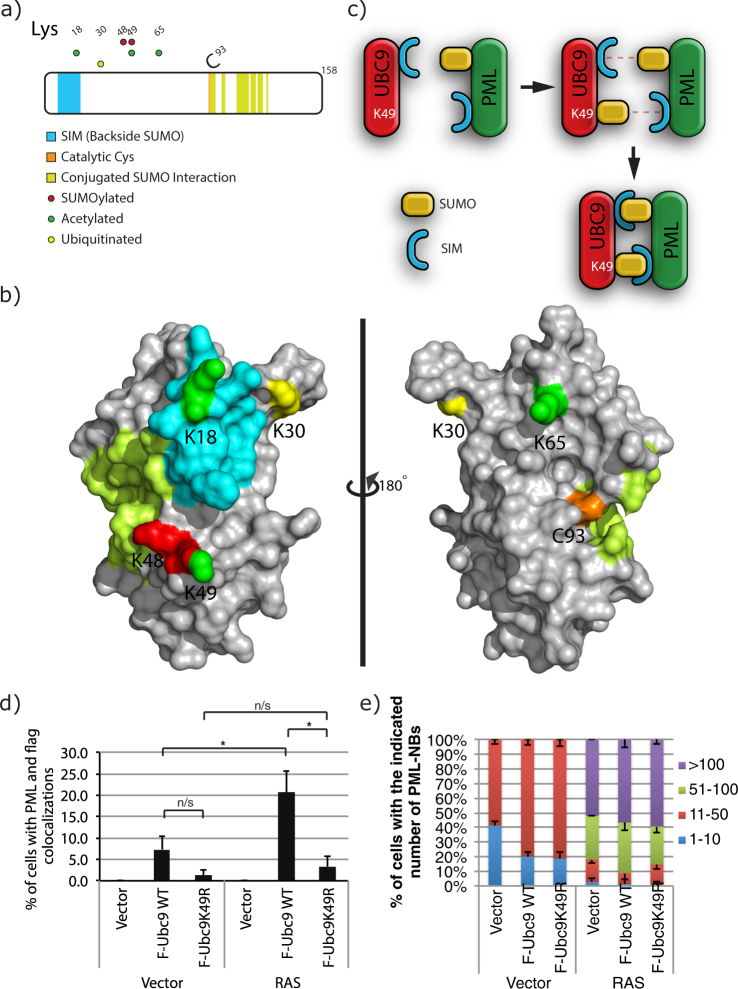
Post-Translational Modifications on UBC9 in U2OS cells Identified by IP and LC-MS/MS. (**a**) UBC9 is a heavily post-translationally modified protein as shown by the distribution of SUMOylation, Ubiquitylation and Acetylation sites identified on UBC9 from our IP and LC-MS/MS experiments. (**b**) Modified sites depicted on the UBC9 crystal structure (3UIP), using the color scheme from (**a**). (**c**) Model of the interaction brought about by the SUMOylation of UBC9 at Lys-49 and PML as an example of a protein with both SUMOylated site and SIM (SUMO interacting motif). (**d**) Quantification for the colocalization of UBC9 and PML were obtained from immunofluorescence analyses using monoclonal mouse anti-Flag and rabbit anti-PML antibodies in U2OS cells stably expressing SUMO3m with wild type UBC9 (F-Ubc9 WT) or its K49R variant (F-Ubc9K49R) and with an empty vector (Vector) or a vector expressing RAS. Cells were fixed for immunofluorescence ten days after transduction. (**e**) Quantification of the number of PML-NBs in U2OS cells when expressing SUMO3m with wild type UBC9 (F-Ubc9 WT) or its K49R variant (F-Ubc9K49R) and with an empty vector (Vector) or a vector expressing RAS.

UBC9, the sole E2 SUMO-conjugating enzyme responsible for all protein SUMOylation in mammalian cells, was found to be more SUMOylated at its Lys-49 in senescent cells. Since it has been extensively documented that lysine residues found to be SUMOylated can often be modified by other PTMs such as acetylation and ubiquitylation^26^, we examined all detectable PTMs for UBC9 during senescence and more specifically the occurrence of modifications at Lys-49. We constructed a Flag-UBC9, and performed anti-Flag IP of extracts followed by LC-MS/MS analysis from U2OS senescent cells (Fig. 4a) and from IMR90 cells (Supplementary Fig. S4a). Our IP LC-MS/MS results garnered several PTMs on UBC9, including the SUMOylation of Lys-48 and Lys-49, acetylation of Lys-18, Lys-49 and Lys-65, and ubiquitylation of Lys-30.

We used the crystal structure of UBC9 (3UIP) to map the identified PTM on the protein surface to gain insights on the potential roles of these modifications on structure-function relationships (Fig. 4b for U2OS and Supplementary Fig. S4b for IMR90). Of all the PTM residues observed, only acetylation of Lys-18 appears to lead to a possible distinguishable function based on known UBC9 domains, since Lys-18 acetylation could affect UBC9’s backside SUMO interaction, which is believed to mediate polySUMO chain formation^37^.

In contrast, based on a general topological level, SUMOylation of Lys-49 could promote a fixed appendage on UBC9 that favors its interaction with SIM containing proteins. Moreover, Lys-49 is located in close proximity to the backside SIM of UBC9. SUMOylation of UBC9 at Lys-49 could not only promote its interaction with SIM containing proteins, but also with proteins that are both SUMOylated, and contain a SIM (Fig. 4c). The latter interaction would provide a mechanism by which bidirectional SUMO-SIM binding generates an interaction that is directional and potentially stronger since it would promote two SIM-SUMO interactions. The cooperativity of multiple SUMO binding interactions has been shown *in vitro* where RNF4 shows a 10-fold increase in affinity for SUMO2 dimers than for SUMO2 monomers^38^. One such candidate protein that is known to have a SIM and be heavily SUMOylated is PML. Indeed, PML is known to be heavily SUMOylated at Lys-65, Lys-160 and Lys-490, and also harbors a SIM at its C-terminus^14,19^. Since most of the senescence regulated SUMO sites are associated with PML-NBs, it is plausible that the SUMOylation of UBC9 could indeed have a role in its PML-NB association. In addition, Lys-49 is on the backside of UBC9 far away from the active site (Cys-93), therefore its SUMOylation should not alter UBC9 catalytic function, suggesting that it may rather play a role in scaffolding/interactions.

Prior to performing in cell assays, we validated that a K49R variant of UBC9 was indeed functional through *in vitro* assays. Time course SUMOylation of RanGap, of a SUMOylatable peptide of PML and of E2–25K showed virtually identical kinetics for the wild type and the K49R variant forms of UBC9 highlighting that the K49R alteration has no effect on the conjugating activity (Supplementary Fig. S5a). To investigate the role of SUMOylation of UBC9 at Lys-49 *in vivo*, we produced cells expressing either a Flag-Ubc9-K49R or a Flag-Ubc9 wild type construct and performed Flag IP to determine if UBC9 SUMOylation was lost in the K49R variant versus the wild type. Western blots of IP material indeed revealed that Lys-49 is a major site of UBC9 SUMOylation by SUMO2/3 in U2OS (Supplementary Fig. S5b). The K49R variant of UBC9 is still SUMOylated suggesting the presence of other SUMO acceptor sites on UBC9 contributing normally to the band shift or compensating for the loss of Lys-49, most likely Lys-48 that we identified in our Flag IP coupled with LC-MS/MS (Fig. 4a). Of note, through these analyses we observed that ~5% of wild type UBC9 proteins were found to be SUMOylated in the cell (Supplementary Fig. S5b and c). This is the upper limit of the stoichiometry of protein SUMOylation considering that SUMOylation is thought to occur at <5%, with the exception of a few proteins, such as RanGAP and PML^39^.

To address our hypothesis that SUMOylation of UBC9 promotes its association with PML (Fig. 4c) and PML-NBs, we opted to use immunofluorescence assays. We found that in U2OS cells the wild type UBC9 colocalized more readily than its K49R variant in both control cells and upon RAS-induced senescence (Fig. 4d and Supplementary Fig. S6). Similar findings were also observed in IMR90 cells (Supplementary Fig. S7a). Importantly, we observed a 3-fold increase in colocalization of Flag-Ubc9 with PML in RAS-induced senescent U2OS cells compared to control cells. This is in perfect agreement with our quantitative SUMO proteomics approach that showed a 3.36-fold increase in SUMO abundance on Lys-49 of UBC9 in senescent cells. Immunofluorescence analysis of U2OS cells that express either Flag-Ubc9 wild type or Flag-Ubc9-K49R constructs revealed that the number of PML-NBs is not affected by the exogenous UBC9 expression when cells have entered the senescent state (Fig. 4e). Under basal conditions the number of PML-NBs are induced to the same degree by either the Flag-Ubc9 wild type or Flag-Ubc9-K49R constructs. Two important pieces of information can be drawn from these results. First, UBC9 does not localize readily or accumulates to PML-NBs under basal conditions (95% of cells don’t present significant colocalization) (Fig. 4d), but rather requires a stimulus. Second, the integrity of Lys-49 is important for UBC9 colocalization to PML-NBs. We suspect that SUMOylation at Lys-49 is responsible for this colocalization though we cannot rule out the possibility that acetylation may play some role since we observed this PTM at Lys-49 in our IP LC-MS/MS results in U2OS cells (Fig. 4a).

### Recruitment of UBC9 to PML-NBs Serves an Anti-Senescent Function

**Figure 5:**
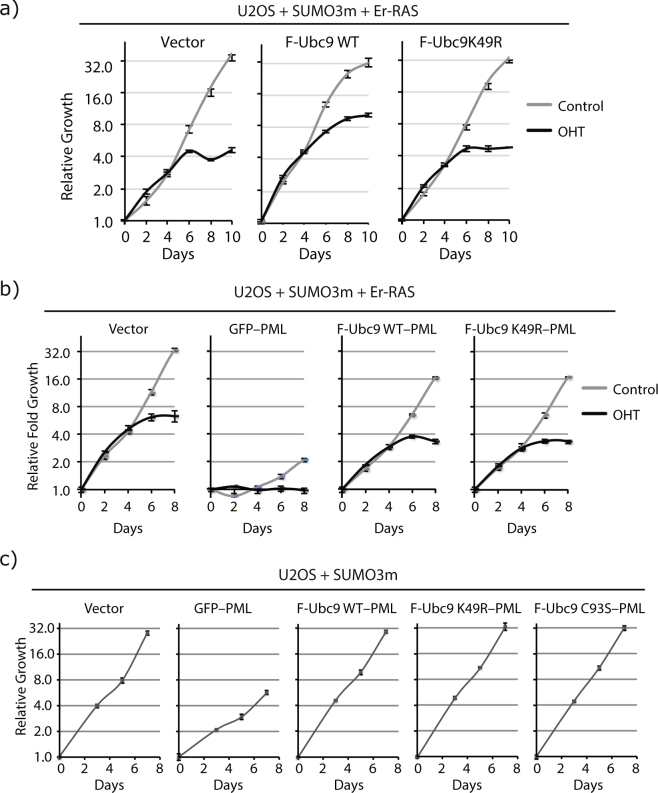
UBC9 Can Exhibit Anti-senescent Properties when Forced to PML-NBs. (**a**) Growth curves of U2OS cells expressing SUMO3m and an Er-H-RAS-G12V construct (fusion of the ligand-binding domain of the estrogen receptor with H-RAS-G12V to control its activity) (Er-RAS) and transduced with a control vector, Flag-Ubc9 wild type (F-Ubc9 WT) or Flag-Ubc9 with the K49R mutation (F-Ubc9K49R). Cells grew normally in the absence of the inducer for Er-RAS (control, grey curves) but enter senescence upon Er-RAS activation with 4-hydroxytamoxifen (OHT, 100 nM changed every two days, black curves). (**b**) Growth curves of U2OS cells expressing SUMO3m and an Er-H-RAS-G12V (Er-RAS) construct and transduced with a control vector, a GFP-PML fusion, a fusion of PML with wild type Ubc9 (F-Ubc9 WT-PML) or its K49R variant (F-Ubc9K49R-PML). Cells were either control treated (grey curves) to observe growth effect of the fusions alone or treated with 4-hydroxytamoxifen (OHT, 100 nM changed every two days, black curves) to observe the effects of the fusion in the context of RAS-induced senescence. (**c**) Growth curves of U2OS cells transduced with SUMO3m and a control vector, a GFP-PML fusion, a fusion of PML with wild type Ubc9 (F-Ubc9 WT-PML), its K49R variant (F-Ubc9K49R-PML) or the catalytic mutant C93S (F-Ubc9C93S-PML).

To further understand the biological role of UBC9 SUMOylation at Lys-49 we studied the effect of the K49R variant on the onset and amplitude of RAS-induced senescence. Figure 5a demonstrates that under basal conditions (gray curves) expression of Flag-Ubc9 wild type or Flag-Ubc9-K49R in U2OS cells has no adverse effect on cellular growth (Supplementary Fig. S7b for IMR90 cells). However, under senescence promoting conditions (when Er-RAS is activated with 4-hydroxytamoxifen (OHT), black curves in Fig. 5a) there is a clear delay for the onset of senescence in U2OS when cells express the wild type form of UBC9 while the K49R variant shows no effect and undergoes senescence like the control cells (Fig. 5a). Interestingly, this phenomenon was not observed in IMR90 cells (Supplementary Fig. S7b). The IMR90 cells that either expressed Flag-Ubc9 wild type or Flag-Ubc9-K49R entered the senescent state with the same dynamics as the control cells that do no express the FLAG-Ubc9 constructs. Of note, expressing the viral E7 protein, which inhibits the RB/E2F pathway, promoted the senescence bypass by wild type Ubc9 (Supplementary Fig. S8). This is in agreement with the wild type UBC9 mediated bypass of senescence in U2OS cells since these cells intrinsically have a defective RB/E2F pathway due to CDKN2A loss of expression.

**Figure 6:**
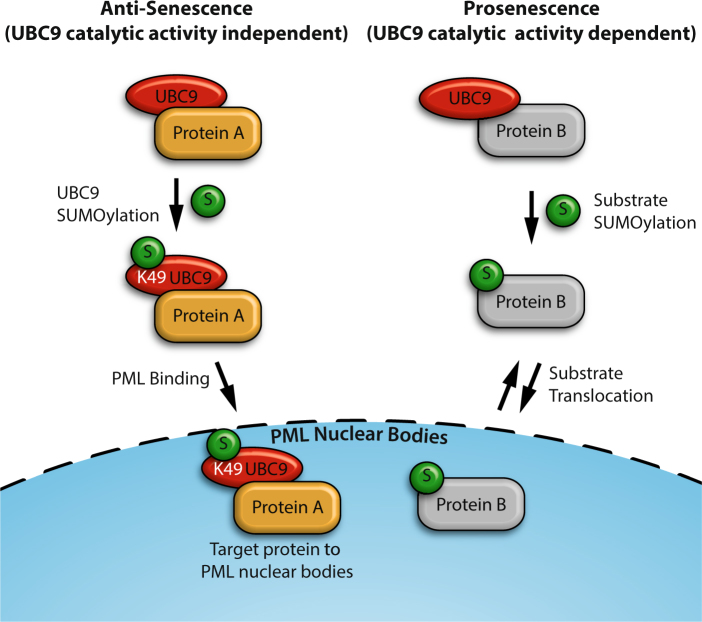
SUMOylation of UBC at Lys-49 favors it relocalization to PML-NBs and promotes the translocation of target protein(s) to the nuclear bodies, conveying an anti-senescence phenotype. Whereas SUMOylation of proteins by the non-SUMOylated UBC9 promotes senescence.

Considering that the K49R alteration has no effect on UBC9 activity (Supplementary Fig. S5a) but hinders its association to PML-NBs (Fig. 4d), these results suggest that SUMOylation of UBC9 at Lys-49 promotes its localization to PML-NBs where it can serve an anti-senescent function. This concept was further validated by studying the effect of expressing Ubc9 wild type and K49R in fusion with PML to force their localization to follow PML in PML-NBs, and evaluate the effect on both PML-induced senescence and RAS-induced senescence. As expected, both Ubc9-PML and Ubc9K49R-PML fusion proteins localized to nuclear bodies (Supplementary Fig. S9). Expressing either Ubc9-PML or Ubc9K49R-PML fusion proteins counteracted the senescence induced by PML as compared to GFP-PML fusion in control cells (gray curves, Fig. 5b). Moreover, cells treated with OHT to activate Er-RAS (black curves, Fig. 5b) entered RAS-induced senescence as before but GFP-PML further strengthen the growth arrest while either Ubc9-PML or Ubc9K49R-PML did not do so even in the context of RAS senescence. Bypass of PML-senescence by fusion with UBC9 is not unique to U2OS cells as we observed similar results in IMR90 cells (Supplementary Fig. S10). Non enzymatic functions of UBC9 have been reported and have shown that UBC9 can, in the absence of its SUMO conjugation activity, promote cell invasion and metastasis^40^. Interestingly, the catalytically inactive form of Ubc9 (C93S variant) when incorporated into the PML fusion construct could also bypass senescence with the exact same dynamics as the wild type and K49R variant of Ubc9 (Fig. 5c). Of note, all the fusion proteins were correctly expressed in the cells and did not produce a change in the global SUMO proteome, which could have caused phenotypic changes to the cells (Supplementary Fig. S11). Thus, the senescence bypass mediated by UBC9 is not due to the SUMOylation of target proteins but rather to another non enzymatic function of UBC9. Overall, these results further support the concept that localization of UBC9 to PML-NBs harbors an anti-senescent property that is independent of UBC9 Lys-49 integrity and independent of its SUMO conjugating activity (Fig. 6). Of note, while GFP-PML fusion induced growth arrest and senescence in U2OS cells, a fusion protein containing GFP and a form of PML that cannot be SUMOylated (where six SUMOylated lysine residues – K65, K160, K380, K478, K490 and K497 – are converted to arginine residues; PML6K) induced a much stronger growth arrest (Supplementary Fig. S12). This senescence phenotype could still be partially rescued with the fusion of Ubc9 (Ubc9-PML6K, Supplementary Fig. S12). These results further demonstrate that SUMOylation of PML itself in cis by UBC9 is not the main event that provides the bypass of the senescent phenotype, but rather relies on the non-enzymatic function of UBC9.

## Discussion

This study presents the first large scale SUMO proteome in cells subjected to RAS-induced senescence. We opted to use the SUMO site identification methodology rather than the classic SUMOylated protein approach, since the added information of SUMO site allowed for easier biological validation of target proteins, namely UBC9 in this study. Moreover, quantifying changes in SUMO sites rather than SUMO proteins allowed to quantify multiple sites on proteins, as observed for TRIM28 and XPC (Table 1), that is otherwise not achievable when we quantify SUMOylated proteins as a whole. The overall number of SUMO sites identified in this study is considerably lower than in our previous articles due to the nature of the treatment under study and possibly due to the cell type. Indeed, normally harsher treatments that increase SUMO conjugated levels by more than 5–fold are studied by proteomics, while the RAS-induced senescence garnered only a minor increase in global SUMOylation as observed in Fig. 1b. Perhaps a more dramatic change in the SUMO proteome could have been observed if an earlier point during the onset of senescence was investigated rather than looking at the final established state. Importantly, 95% of the SUMO sites identified in this study had already been reported in the literature, highlighting the validity of the data presented here (Supplementary Fig. S13)^41^. On the other hand, the overlap of the SUMO sites identified in this study to those identified endogenously with the WaLP protease was 18%^42^. This is similar to the overlap observed with all the large scale proteomics studies combined and the sites observed with the WaLP procedure. Indeed, this poor overlap was attributed to large differences between the WaLP procedure and the standard SUMO proteome studies where different peptide pools are generated due to the different proteases employed.

Despite detecting a moderate increase in the intensity of the SUMOylated bands observed by western blot for the senescent cells (Fig. 1b), the proteomics data revealed that only a few targets had increased SUMOylation levels (Table 1). Although poly-SUMOylation (SUMO2 and SUMO3, Table 1) could explain some of the changes seen on western lot, there was otherwise roughly the same number of up- and down-regulated SUMO sites upon RAS-mediated senescence. This leads to the notion that performing SUMO2/3 blots to study the effect of a stressor may not be ideal and that an in depth analysis should be adopted.

The majority of the senescence regulated SUMO sites were from proteins associated to PML-NBs (Fig. 3a). This is in line with their increased number and size in senescent cells (Figs 3b and 4e). We also studied the localization of HSPA5, a protein known to localize to the endoplasmic reticulum, and found the protein to be localized in part in PML-NBs upon senescence (Fig. 3c). This observation strengthens the notion that proteins that are not considered residents of PML-NBs could localize to this nuclear sub compartment under certain conditions. Therefore, it is likely that more proteins from our regulated list are localized in PML-NBs during senescence.

We found that SUMOylation of UBC9 at Lys-49 is more present in senescent cells (Fig. 2e) and showed that the integrity of this site is important for UBC9 association to PML-NBs (Fig. 4d, Supplementary Figs. S6 and S7a). This is reminiscent of our observations with the proteasome in HEK293 cells, where SUMOylation of members of the proteasome promoted its association to PML-NBs in a SIM dependent manner^32^. The mode by which the interaction between UBC9 and PML occurs could deviate from the simple and classic single SUMO-SIM interaction. From the crystal structure of UBC9, we propose that the UBC9/PML interaction is bridged by two SUMO-SIM interactions, where each protein has a SIM and a SUMO, making this binding stronger but also directional (Fig. 4c). There is a growing body of research that describes PML as a genuine SUMO E3 ligase^35,43^. This leads to the idea that dual SUMO-SIM interaction may not be unique to UBC9 and PML. Indeed, most SUMO E3 ligases are SUMOylated and harbor SIM motifs (CBX4, PIAS1, PIAS2, PIAS3, PIAS4, RANBP2 and NSE2)^26–29^. Whether these E3 ligase properties promote their interactions with UBC9 or PML and PML-NBs remains to be evaluated.

We also determined that in U2OS cells the overexpression of wild type UBC9 produced a delay on the onset of RAS senescence whereas the overexpression of the K49R variant entered senescence with the same dynamic as the control cells (Fig. 5a). This observation along with the localization of SUMOylatable UBC9 to PML-NBs (Fig. 4d) suggest that UBC9 in PML-NBs might have a different role during senescence than UBC9 localized in other cellular regions. Considering that forcing WT UBC9, its K49R or its C93S variant into PML-NBs when expressing them as Ubc9-PML fusion can bypass senescence in U2OS-SUMO3m cells, we propose that a function that is independent of the catalytic activity of UBC9 at nuclear bodies inhibits senescence (Fig. 6). The concept of PML-NB associated UBC9 performing an anti-senescent function that is independent of its SUMOylation activity is in agreement with the literature considering that SUMOylation is thought to promote senescence, while down-regulating UBC9 also leads to the same phenotype^44^. Indeed, Bischof *et al*. have shown that activating the SUMO conjugation machinery by overexpressing the PIAS4 SUMO E3 ligase induces senescence in a p53 dependent manner^45^. Moreover, overexpression of PML is known to promote senescence by promoting the SUMOylation of p53 by SUMO1^46^. Conversely, inhibiting the SUMO deconjugation machinery by knocking down SENP1 also leads to a senescent phenotype in a p53 related manner^47^. PML has been implicated in the repression of multiple E2F target genes during senescence^48^ and SUMOylation of proteins in chromatin helps to repress multiple genes during senescence^44^. Lastly, the western blots depicted in Fig. 1b indicated that global changes and increases in SUMOylation and/or poly-SUMOylation on nuclear proteins do occur during senescence.

We have shown that SUMOylation of Lys-49 of UBC9 (a small fraction of the overall UBC9) favors its localization to PML-NBs and that favoring or forcing UBC9 to PML-NB can delay or oppose the onset of senescence (Fig. 5). Moreover, we showed that the catalytically inactive form of UBC9 could promote the same senescence bypass, indicating that the bypass is not mediated by SUMOylation of PML-NB resident proteins. To reconcile these findings we propose the model depicted in Fig. 6. The model stipulates that UBC9 SUMOylated at Lys-49 localizes to the PML-NBs and promotes the translocation of key interacting protein(s) during senescence, thereby delaying the phenotype. The anti-senescence function of UBC9 is consistent with its role in supporting transformation by oncogenic RAS^49^. Of note, it is also intriguing that during RAS-induced senescence we found a decrease in the SUMOylation of TRIM28 while Luo and colleagues found an increase in the SUMOylation of this protein during RAS-driven oncogenic transformation^49^. TRIM28 is degraded by the ubiquitin-proteasome pathway during RAS senescence in normal cells^30^ suggesting that defects in TRIM28 ubiquitination may explain its accumulation in tumor cells.

The role of SUMOylation on the onset of senescence has been documented to be linked to p53 activation, but we were unable to either quantify or identify SUMOylation of p53 by SUMO3 in our proteomic dataset^46,50^. We did however notice some proteins whose levels and SUMOylation occupancies were affected by the senescent phenotype that are linked to p53 activation/inactivation. We observed a decrease in both TRIM28 protein and SUMOylation levels in response to senescence. TRIM28 is known to interact with the E3 ubiquitin ligase MDM2 and promote the ubiquitylation and degradation of p53^51^. Hence, lower levels of TRIM28 may limit the degradation of p53 during senescence. Also, TRIM28 depletion was reported to increase the levels of PML-NBs, which are known to stabilize p53 through phosphorylation and acetylation events^36,52,53^. Moreover, we observed increased levels of SUMOylation of MORC3 in senescent cells, a protein known to promote p53-mediated senescence^54^.

Induction of senescence relies not only on p53 activation but also on the RB/E2F pathway. Work from the DeJean group highlighted that SUMOylation of RB1 and E2F factors are preferentially modified by SUMO2/3 during senescence reprogramming^44^. Moreover, silencing UBC9 caused cells to undergo arrest in a senescence-like state, supporting the notion that UBC9 can serve an anti-senescent role. We found that increasing the levels of WT UBC9 could delay senescence reprogramming in the RB/E2F altered U2OS cells, while this could not be achieved with the non-SUMOylatable form of UBC9 (K49R) (Fig. 5). Interestingly, senescence bypass could not be observed in the IMR90 cells that have an intact RB/E2F pathway (Supplementary Fig. S7b). Rather, the bypass of the senescence reprogramming in IMR90 cells could be achieved by hindering the RB/E2F pathway by introducing the viral oncoprotein E7 (Supplementary Fig. S8). Therefore, SUMOylation of UBC9 at Lys-49 can promote the bypass of the senescence program in cells with a disabled RB/E2F pathway, in line with the work from the group of DeJean^44^ and previous work from the Ferbeyre laboratory^55^. Of note, in their publication the DeJean’s group demonstrated that UBC9 depleted cells possessed a 3-fold increase in RAS levels in the cells, demonstrating an interconnectivity between UBC9 levels and RAS production.

Overall, we report changes in SUMOylation levels on various substrates during senescence using a proteomic approach. Indeed, we found 25 regulated sites and uncovered the unexpected SUMOylation of UBC9 at Lys-49 during senescence. Future studies are required to determine the effect of SUMO1 in senescence since this paralog is thought to be different than SUMO2 and SUMO3. Moreover, the action of SUMO1 on p53 as a senescence activator has been documented, leading to the possibility that other substrates are modulated by this paralog during cell cycle arrest^46^. Considering that HDAC1 SUMOylation levels were regulated during senescence it is possible that acetylation may have important implications on the senescent phenotype, and the further understanding of PTM cross-talk will yield a more in depth knowledge of processes mediating this kind of cell cycle arrest. Ultimately, this knowledge will allow for the development of drugs or agents that can promote senescence and aid in cancer treatments.

## Methods

### Cell culture

U2OS cells were purchased from American Type Culture Collection (ATCC), IMR90 cells were obtained from the Coriell Institute and Phoenix ampho packaging cells were a kind gift from S.W. Lowe. All cells were cultured in Dulbecco’s modified Eagle medium (DMEM; GIBCO) supplemented with 10% fetal bovine serum (FBS; Wisent), 2 mmol/L L-glutamine (Wisent) and 1% penicillin/streptomycin sulfate (Wisent). Retroviral-mediated transductions were performed as described previously^9^.

### Purification of SUMOylated peptides from U2OS cells

U2OS cells stably expressing SUMO3m (6xHis-SUMO3-Q87R-Q88N) were collected by trypsinisation and lysed in hypotonic buffer (10 mM Tris-HCl, pH 7.6, 1.5 mM MgCl_2_, 20 mM 2-chloroacetamide, phosphatase inhibitors and proteases inhibitors) on ice for 30 min. The nuclear fractions were pelleted and washed with hypotonic buffer and the nuclear fraction was pelleted again. Buffer A (6 M guanidinium HCl, 0.1M NaH_2_PO_4_, 10 mM Tris-HCl, pH 8, 10 mM imidazole and 10 mM β-mercaptoethanol) was added to the pellet, sonicated, and centrifuged at 16,000 g. 1 mg of material was set aside for the proteome analysis. 1 mL of 50% Ni-NTA slurry that was pre-equilibrated with buffer A was added for every 25 mg of nuclear material. The tubes were gently rotated at 4 °C for 16 h. The beads were washed once with buffer A, 4 times with buffer B (8M urea, 0.1M NaH_2_PO_4_, 10 mM Tris–HCl, pH 6.3, 10 mM β-mercaptoethanol, 20 mM imidazole) and once with 50 mM ammonium bicarbonate. The proteins were reduced with 5 mM tris(2-carboxyethyl)phosphine (TCEP) for 30 min at 37°C and alkylated with 20 mM 2-chloroacetamide for 30 min at room temperature. Sequencing grade trypsin was added to the beads and allowed to digest 16 h at 37°C.

After digestion, the peptide samples were acidified by adding trifluoroacetic acid to 1% and desalting on HLB cartridges (Waters) prior to lyophilization in a Speed Vac. Peptides were reconstituted in 500 μL of 50% glycerol in PBS. α-NQTGG antibody cross-linked to magnetic protein A/G beads (EMD Millipore) was added to the peptide solution at a 1:2 (w:w; antibody:peptide). The tubes were agitated by inversion for 1 h at 4°C. The tubes were then placed on a magnetic rack, allowing for the beads to settle again and the solution removed. The antibody linked beads were washed 10 times with 1 mL of PBS, once with 1 mL 0.1 X PBS, once with 1 mL of water and eluted with 3 consecutive portions of 100 μL of 0.2% formic acid. The eluents were combined and lyophilized to dryness. The SUMO peptides were reconstituted in 25 μL of 0.2% formic acid in water for analysis on the MS.

### Proteome Sample

500 μg of nuclear-enriched proteins were reduced, alkylated, digested and desalted as for the SUMO peptide preparation. 150 μg of peptides were fractionated using basic porous graphite chromatography. Briefly, peptides were solubilized in PGC-A (5 mM triethylamine formate in water) at 10 μg/μL and resolved on a PGC column using the following program: 0–5% PGC-B (5 mM triethylamine formate in 95% acetonitrile) over 5 min, 5–40% PGC-B over 50 min, and 40-100% PGC-B over 5 min. 81 fractions of 200 μL were collected over the course of the chromatography. The fractions were pooled in the following order: A:8–24; B:25–32; C:33–40; D:41–48; E:49–53; F:54–64. Fractions A-F were lyophilized in a Speed Vac and resuspended in 250 μL of 0.2% formic acid in water for MS analysis.

### LC-MS/MS analyses

A Proxeon EASY-nLC system connected to either a Fusion or Q-Exactive mass spectrometer (Thermo Fisher Scientific) operated in positive ion mode was used for all experiments. 10 μL of each sample was injected on a reverse-phase pre-column (5 mm length, 360 µm i.d.) and separated on a reverse-phase analytical column (18 cm length, 150 µm i.d.) (Phenomenex). Both columns were manually packed in-house. LC were run at a flow rate of 0.6 μL/min using a linear gradient of 5–30 % aqueous acetonitrile (0.2% formic acid) over 56 minutes for the proteome samples or over 106 minutes for the SUMO peptides.

MS survey scans were performed at a resolution of 70,000 at m/z 200 with a mass window of m/z 350-1,500, maximum injection time of 200 ms and an automatic gain control of 1e6. MS/MS scans were acquired using a data dependent acquisition approach with a Top12 method for the proteome or Top speed of 3 s for SUMO peptides. The precursor isolation window was set to 2 m/z with a HCD normalized collision energy of 25, and a resolution of 17,500 at m/z 200. Automatic gain control (AGC) target values for MS/MS scans were set to 2e5 with a maximum fill time of 60 ms for proteome samples or an AGC of 5e3 with a maximum fill time of 3 s for SUMO peptides. A dynamic exclusion of the previously acquired precursor ions was set to 15 s.

### Mass spectrometry data processing and statistical analysis

MS/MS spectra were searched against Uniprot/SwissProt database including Isoforms (released on February, 2013) using MaxQuant (version 1.5.1.2)^56,57^. The first search peptide tolerance was set to 20 ppm, the main search to 10 ppm, and fragment ion tolerance to 7.5 ppm since all ions were analyzed in the orbitrap. The maximum allowed number of missed cleavage is set to 3 using trypsin as enzyme with a maximum of 5 modifications per peptide. For SUMO peptide searches carbamidomethylation of cysteine residues was set as a fixed modification, while methionine oxidation, asparagine and glutamine deamidation, lysine SUMO3(NQTGG)^58^, protein N-acetylation, Lysine-GlyGly, and Phospho (STY) were set as variable modifications. For proteome searches carbamidomethylation of cysteine residues was set as a fixed modification, while methionine oxidation, asparagine and glutamine deamidation, and protein N-acetylation were set as variable modifications. The false discovery rate (FDR) for peptide and proteins were set to 1%, and the minimum peptide length was set to 6.

The MaxQuant output files were processed using the Perseus software (version 1.5.0.8). SUMO site identification were filtered by removing all “reverse” sites from the list. Furthermore, the SUMO site list was filtered with a SUMO site probability score of 0.75 or greater, which is routinely used for PTM site identification methodologies. The reported SUMOylated peptide intensities were Log2 transformed for each of the 5 biological replicates. SUMO sites that were present in at least 3 of the 5 biological replicates were retained for further processing. The intensity of the various replicates were normalized by subtracting the median intensity from each experiment. Imputations were employed for SUMO sites that were quantified in only one condition (present in RAS only or present in control samples only) using normally distributed values with a randomized 0.3 width (log2) and a 1.8 down shift (log2). SUMO sites were deemed statistically regulated by RAS if the 2 sample T-test p-values were ≤0.05. For the proteome analysis, a more stringent filtering was conducted. Proteins that were quantified in at least 4 of 5 replicates and a permutation-based FDR of less than 5% was considered as significantly regulated.

### Bioinformatic analyses

The global SUMO motif was generated using a 31 amino acid sequence window centered about the target Lys residue. Motifs were extracted using pLOGO v1.2.0 at https://plogo.uconn.edu/^59^. STRING networks were generated using STRING database^60^. Cytoscape 3.2.0 was used to visualize the network^61^. Gene ontology term enrichments were performed using Database for Annotation, Visualization and Integrated Discovery^62^. The following terms were analyzed versus the human proteome: Biological Processes, Molecular Functions, and Cellular Compartments.

### *In vitro* SUMOylation Assay

SAE1/SAE2, Mg-ATP, E2-25K used in the *in vitro* SUMO assay were obtained from Boston Biochem (Cambridge, MA). GST-RanGAP fragment 418–587 and GST-PML fragment 485–495 were purchased from Biomol International (Farmingdale, NY). MmUbc9 WT and K49R variant proteins were expressed and purified from the pET28a backbone as described earlier^63^. *In vitro* reactions were composed of 0.5 µM substrate (RanGAP, PML and E2–25K), 12.5 µM His_6_-SUMO3 Q92R, 65 nM SAE1/SAE2 and 0.5 µM wild type or K49R Ubc9 in activity buffer (20 mM HEPES (pH 7.8), 50 mM NaCl and 1 mM DTT). The samples were incubated at 37°C for 10 min before the addition of MgATP to 5 mM (except for –ATP reactions where MgATP was omitted). At each time point, 5 µL (2.5 pmol of substrate) were withdrawn from the reaction mixture and placed into 10 µL of 1.5X laemmli buffer.

### Western blotting

Protein extracts were boiled for 10min in Laemmli buffer (10% (w/v) glycerol, 2% SDS, 10% (v/v) β-mercaptoethanol and 62.5 mM Tris–HCl, pH 6.8) and separated on a 4–12% SDS–PAGE followed by transfer onto nitrocellulose membranes. Prior to blocking the membrane for 1 h with 5% non-fat milk in TBST, membranes were briefly stained with 0.1% Ponceau-S in 5% acetic acid to represent total protein content. Membranes were subsequently probed with the indicated primary antibody in blocking solution for 16 h at 4°C: (E2-25K, 1:2000, ab52930, Abcam; GST, 1:500, ab9085, Abcam; HDAC1 was a kind gift from Dr, Alain Verreault; HIS-tag, 1:5000, 631212, Clontech; Histone H3, 1:1000, 9715, Cell Signaling; Histone H4, 1:1000, 2592, Cell Signaling; PML, 1:200, H-238, Santa Cruz; RAS, 1:1000, 3965, Cell Signaling; SP100, 1:300, ab43151, Abcam; SUMO2/3, 1:2000, 51–9100, Zymed; Tubulin, 1:1000, 2144, Cell Signaling; UBC9, 1:1000, 33044, Abcam; TRIM28/KAP-1, 1:200; A300-274A, Bethyl Laboratories.) The membranes were incubated with secondary antibodies (goat anti-rabbit HRP, AP307P, EMD Millipore, 1:5000 and goat anti-mouse HRP, AP308P, EMD Millipore, 1:5000) for 1 h. Membranes were washed three times with TBST. Membranes were revealed using ECL (GE healthcare) as per the manufacturer’s instructions, and chemiluminescence was captured on Blue Ray film (VWR) or with a BioRad ChemiDoc MP Imaging System.

### Immunoprecipitation

Frozen cells collected by trypsinisation from 4 x 15 cm petri were defrosted for 30 min on ice in LSB buffer (10 mM Tris-HCl pH 7.6, 1.5 mM MgCl_2_, 20 mM 2-chloroacetamide, protease inhibitors (Sigma), 1 mM Na_2_MoO_4_, 1 mM Na_3_VO_4_ and 4 mM sodium tartrate). Samples were briefly vortexed and nuclei pelleted. The nuclear pellets were washed with LSB buffer and pelleted again. The nuclei were resuspended in Extraction buffer (50 mM Tris-HCl pH 7.6, 1.5 mM MgCl_2_, 420 mM NaCl, 420 mM EDTA, protease inhibitors (Sigma), 1 mM Na_2_MoO_4_, 1 mM Na_3_VO_4_ and 4 mM sodium tartrate) and sonicated for 5 seconds. Flag-agarose slurry (Sigma) were prewashed 4 times with Extraction buffer and added to the protein samples. Volumes were adjusted to 1 mL with Extraction buffer. Samples were mixed under rotation for 2 h at 4°C. The beads were washed 3 times with ice cold Extraction buffer followed by a wash with 1 mL of PBS. Immune complexes were eluted with three sequential incubations with Elution buffer (6 % ammonium hydroxide in water, pH 11–13). Eluates were combined and lyophilized to dryness in a speed vacuum. For LC-MS/MS, samples were reconstituted in 25 μL of 0.2% formic acid in water and injected as indicated above. For western blot analysis, samples were resuspended in 100 μL of Laemmli sample buffer (60 mM Tris-HCl pH 6.8, 2 % SDS and 10 % glycerol) and 20 μL were loaded on a 4–12% SDS-PAGE.

### Fluorescence microscopy

Immunofluorescence experiments were performed as previously described^64^. In short, cells were grown on coverslips for at least 16 h before fixing with 4% paraformaldehyde in PBS for 10 min at 4 °C. In the case of SP100 and HSPA5 immunofluorescence, cells were pre-incubated in CSK buffer (25 mM HEPES, 50 mM NaCl, 1 mM EDTA, 3 mM MgCl_2_, 300 mM sucrose). The cover slips were then washed with PBS and the cells were permeabilized with 0.2% Triton X-100 in PBS/BSA 3%. Cells were then washed in PBS/BSA 3% and incubated overnight with the desired combination of the following primary antibodies: anti-HSPA5/GRP78 (1:100, SPA-826; Stressgen), anti-PML (1:600, G-8, SantaCruz Biotechnology, or 1:600, A301-167A, Bethyl Laboratories), anti-FLAG M2 tag (1:400; F1804, Sigma-Aldrich), anti-UBC9 (1:150, 4786, Cell Signaling). The cells were then washed three times with PBS/BSA 3% and incubated with the secondary antibody of choice (1:4000, AlexaFluor 488 goat anti-mouse, AlexaFluor 488 goat anti-rabbit, AlexaFluor 568 goat anti-mouse, or AlexaFluor 568 goat anti-rabbit; Molecular Probes-Invitrogen) for 1 h at room temperature. Finally, cells were washed three times with PBS and mounted on slide with Vectashield Antifade Mounting Medium containing 1.5 μg/mL of DAPI DNA counterstain. Images were captured with an Axio-Image Z2 microscope from Zeiss or with a super resolution microscope Axio Observer Z1 Elvyra PS.1 from Zeiss for HSPA5. For super resolution structured illumination microscopy, image sets of 5 subsets were taken each after rotating the grid by 5 degrees. A high-resolution image was extracted from the raw data using the Structures Illumination Microscopy and the Maximum Intensity Projection algorithms at a lateral resolution (XY) of 120 nm and an axial resolution (Z) of 300 nm.

### Data availability statement

The raw data for SUMO site identification and quantification that support the findings of this study are available from Peptide Atlas, http://www.peptideatlas.org with the accession code PASS01172 using the following password: grthibault. The additional data that support the findings of this study are available from the corresponding author on request.

## Electronic supplementary material

Supplementary Figures

Supplementary Dataset 1

Supplementary Dataset 2
