# Supplementary material for: Quantitative SUMO proteomics reveals the modulation of several PML nuclear body associated proteins and an anti-senescence function of UBC9
Source: Sci Rep. 2018 May 17;8:7754. doi: 10.1038/s41598-018-25150-z (PMC5958138; doi:10.1038/s41598-018-25150-z)
Supplement: Supplementary file 1 — Supplementary Figures [file 41598_2018_25150_MOESM1_ESM.docx]

**Supplementary information**

**Quantitative SUMO proteomics reveals the modulation of several PML nuclear body associated proteins and an anti-senescence function of UBC9**

Francis P. McManus,^1^ Véronique Bourdeau,^2^ Mariana Acevedo,^2^ Stéphane Lopes-Paciencia,^2^ Lian Mignacca,^2^ Frédéric Lamoliatte,^1,3^ John W. Rojas Pino^2^, Gerardo Ferbeyre^2*^ and Pierre Thibault^1,3*^

^1^Institute of Research in Immunology and Cancer, ^2^Department of Biochemistry and Molecular Medicine, ^3^Department of Chemistry, Université de Montréal, Montréal, QC H3C 3J7, Canada

***Correspondence:**

Pierre Thibault, Phone: (514) 343 6910, E-mail: pierre.thibault@umontreal.ca

Gerardo Ferbeyre, Phone: (514) 343 7571, E-mail: [g.ferbeyre@umontreal.ca](mailto:g.ferbeyre@umontreal.ca)

**Supplementary Materials:**

| **Supplementary Table S1** | Statistics on the Identification and Quantification of SUMO Sites. |
| --- | --- |
| **Supplementary Table S2** | Statistics on the Identification and Quantification of Proteins. |
| **Supplementary Data S1:**  **Supplementary Data S2:** | List of SUMO sites identified. (separate excel file)  List of Senescence Regulated Proteins. (separate excel file) |
| **Supplementary Figure S1** | Changes in mRNA Levels Induced by RAS Overexpression for Key Markers of Senescence. |
| **Supplementary Figure S2** | Volcano Plot of Quantified SUMO Sites and Motif Analysis of Identified SUMO sites. |
| **Supplementary Figure S3** | Volcano Plot of Quantified Proteins and their Associated GO Terms. |
| **Supplementary Figure S4** | Post-Translational Modifications on UBC9 in IMR90 cells. |
| **Supplementary Figure S5** | SUMOylation of UBC9 Occurs Primarily at Lys-49 and does not affect its Catalytic Activity. |
| **Supplementary Figure S6** | Immunofluorescence of F-UBC9 WT Co-localizing into PML-NBs More Readily than F-UBC9-K49R Upon RAS Mediated Senescence. |
|  |  |
| **Supplementary Figure S7** | SUMOylation of UBC9 at Lys-49 Promotes its Association to PML-NBs but does not Delay Senescence in IMR90 Cells. |
| **Supplementary Figure S8** | SUMOylation of UBC9 at Lys-49 Bypasses Senescence in IMR90 Cells when the RB/E2F pathway is Inhibited. |
| **Supplementary Figure S9**  **Supplementary Figure S10** | PML Fusion Proteins Localize to PML-NBs.  UBC9 Provides Anti-senescent Properties when Forced to PML-NBs in IMR90 cells. |
|  |  |
| **Supplementary Figure S11** | Ubc9-PML Fusion Protein Expression do not Increase the SUMO Proteome of the Cells. |
|  |  |
| **Supplementary Figure S12** | UnSUMOylatable PML induces senescence but its fusion with Ubc9 bypasses the phenotype. |
| **Supplementary Figure S13** | Overlap of SUMO Sites Identified in this Study to those Identified in Past Large Scale Proteomic Studies and those Identified Endogenously Using the WaLP Procedure. |
| **Supplementary Figure S14**  **Supplementary Figure S15**  **Supplementary Figure S16** | Uncropped Western blots for Figure 1.  Uncropped Western blots for Figure 2.  Uncropped Western blots for Supplementary Figure S5. |

## Supplementary Table S1: Statistics on the Identification and Quantification of SUMO Sites.

|  | Control | RAS | All |
| --- | --- | --- | --- |
| # SUMO Sites Identified | 181 | 243 | 266 |
| # SUMO Sites Identified with Localization Probability > 0.75 | 171 | 228 | 244 |
| # SUMO Sites Quantified in ≥ 60 % of replicates | 90 | 93 | 108 |
| # Up Regulated SUMO Sites (RAS/Control) |  |  | 13 |
| # Down Regulated SUMO Sites (RAS/Control) |  |  | 12 |

## Supplementary Table S2: Statistics on the Identification and Quantification of Proteins.

|  | Control | RAS | All |
| --- | --- | --- | --- |
| # Proteins Identified | 4373 | 4385 | 4714 |
| # Proteins Quantified in ≥ 80 % of replicates | 1738 | 1662 | 2368 |
| # Up Regulated Proteins (RAS/Control) |  |  | 16 |
| # Down Regulated Proteins (RAS/Control) |  |  | 49 |

## *
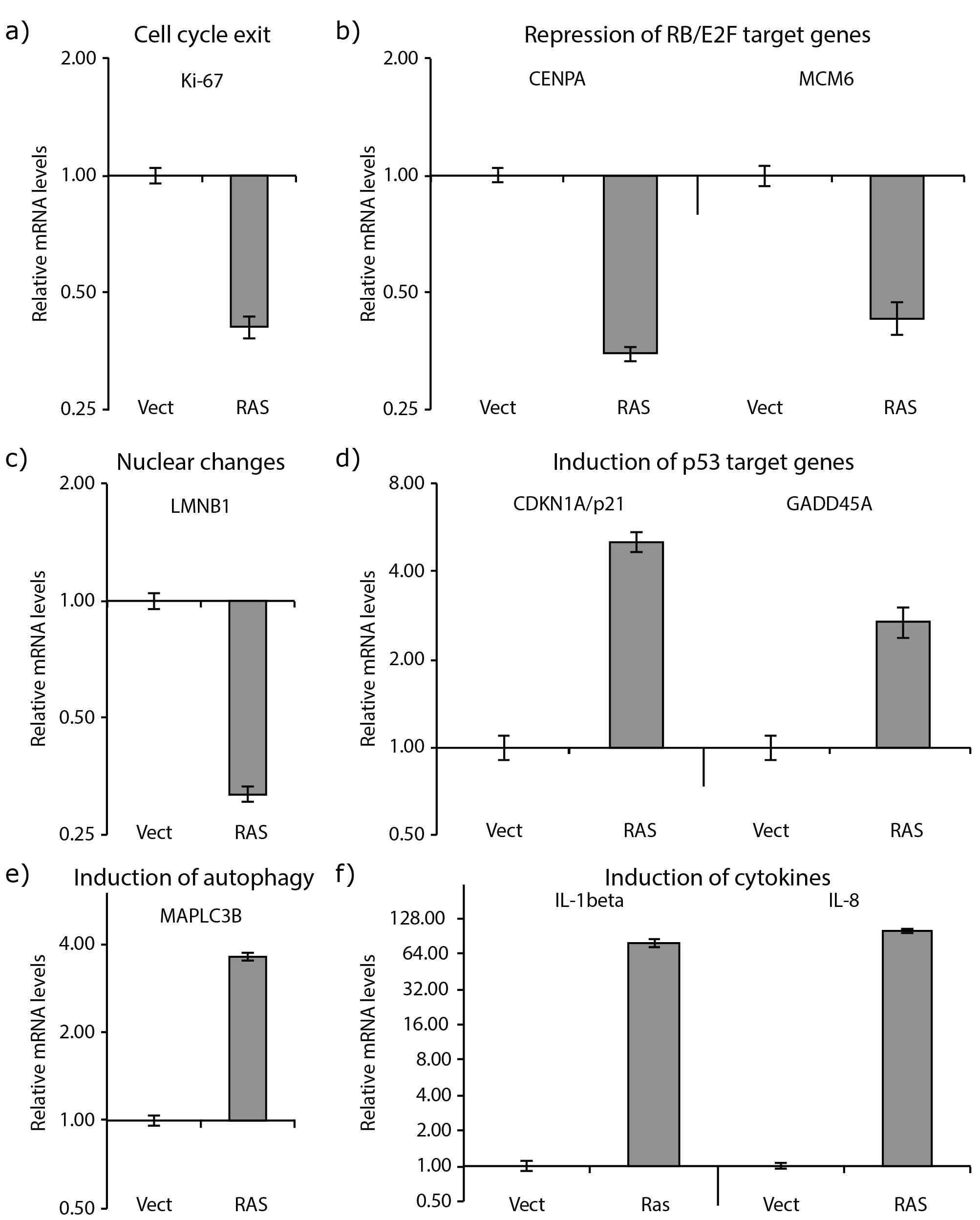
*

## *Supplementary Figure S1: Changes in mRNA Levels Induced by RAS Overexpression for Key Markers of Senescence.*

Relative mRNA levels in U2OS cells expressing SUM3m and a control vector (Vect) or RAS as assed by qPCR for genes involved in (a) Cell cycle exit, (b) Repression of RB/E2F target genes, (c) Nuclear changes, (d) Induction of p53 target genes, (e) Induction of autophagy and (f) Induction of cytokines.


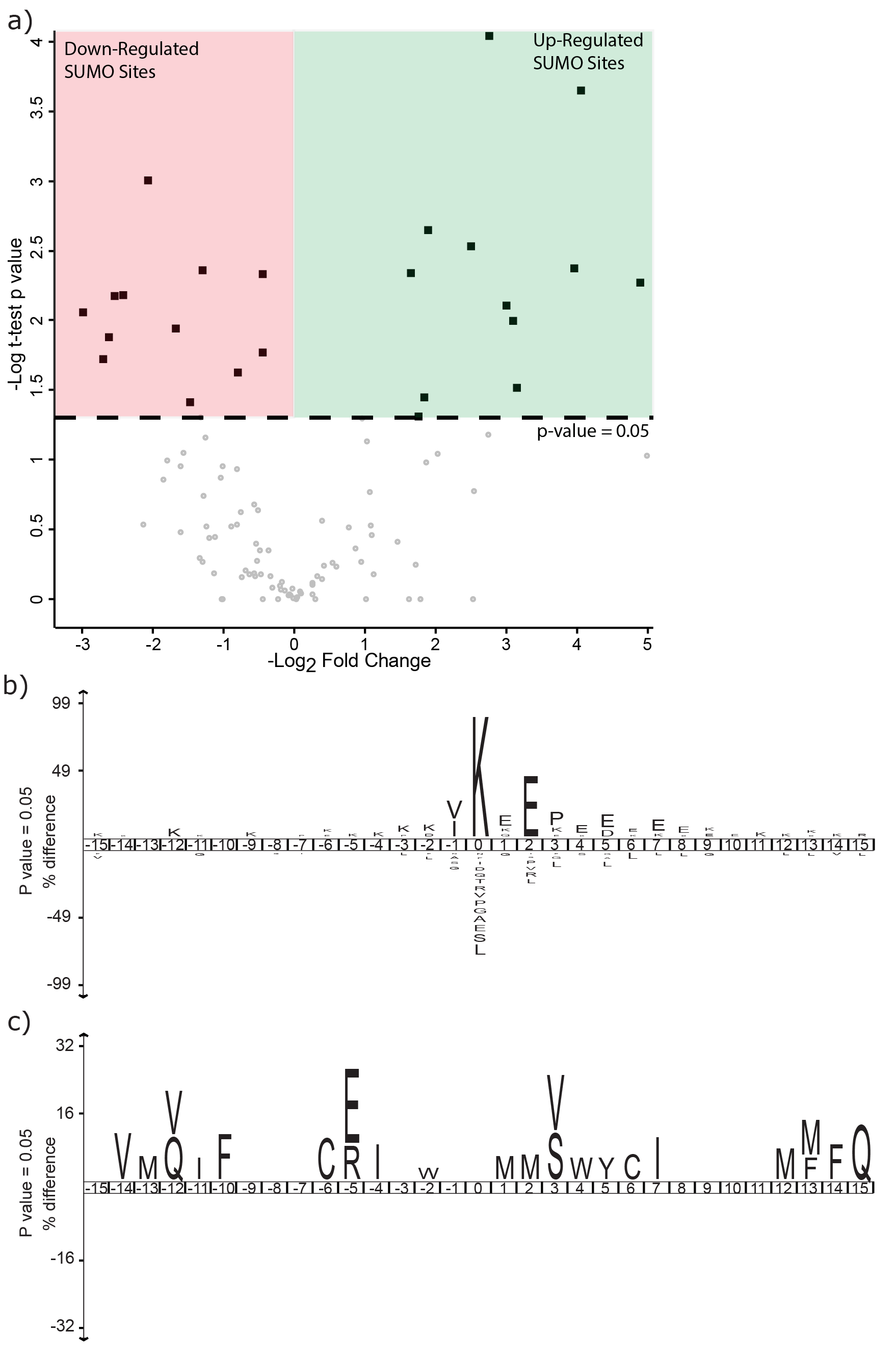


## *Supplementary Figure S2: Volcano Plot of Quantified SUMO Sites and Motif Analysis of Identified SUMO sites.*

(a) Volcano plot of the SUMO site abundance in senescent cells over control cells where each data point represents a site. Sites in the green quadrant are up regulated and in the red quadrant are down regulated in senescent cells. (b) pLogo analysis of all identified SUMO sites. (c) pLogo analysis of the regulated SUMO sites compared to all the sites identified in this study.


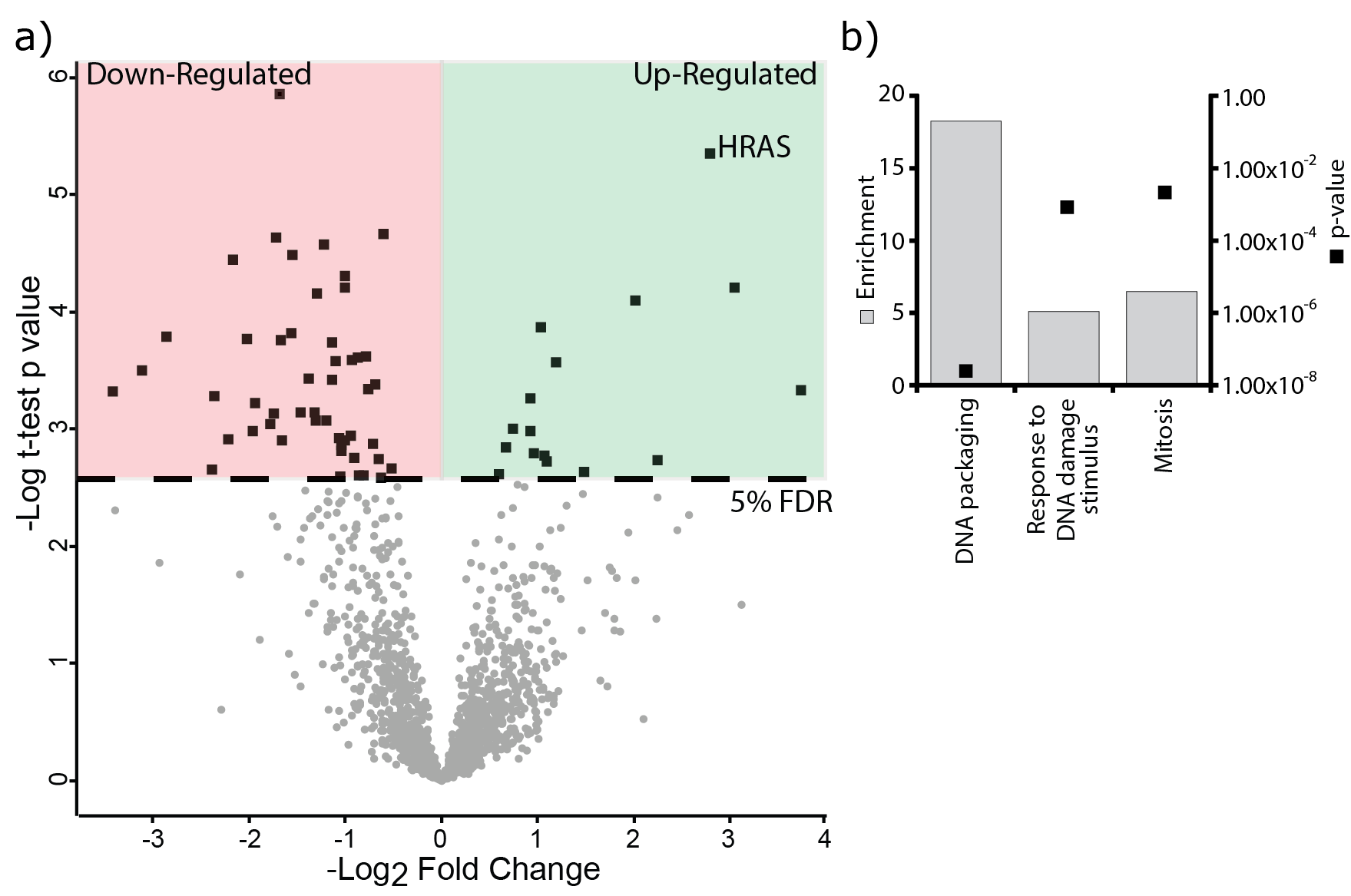


***Supplementary Figure S3: Volcano Plot of Quantified Proteins and their Associated GO Terms.***

(a) Volcano plot of the protein abundance in senescent cells over control cells where each data point represents a protein. Proteins in the green quadrant are up regulated and in the red quadrant are down regulated in senescent cells. (b) GO term associated with the regulated proteins found in the red and green quadrants of panel (a).


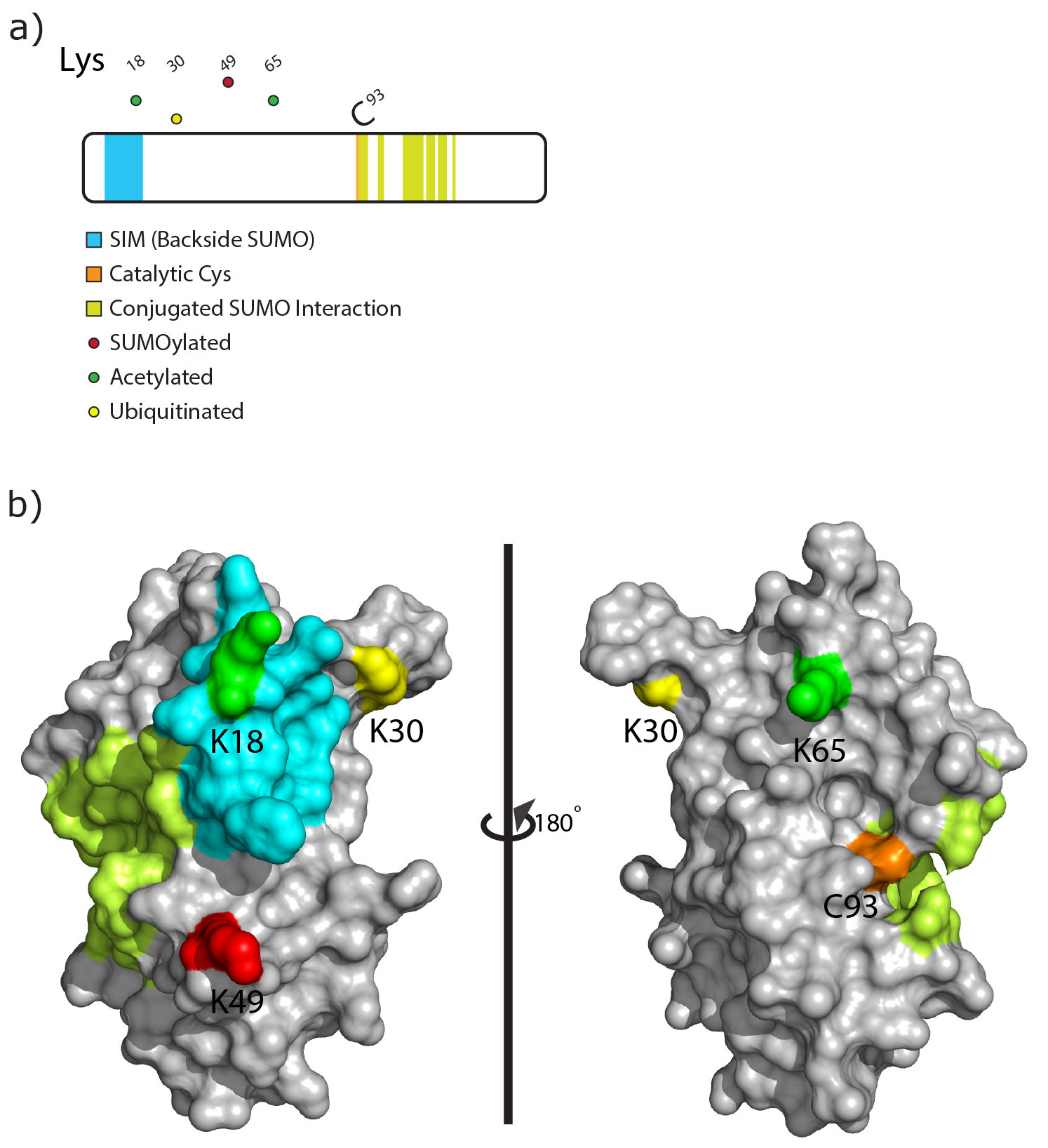


***Supplementary Figure S4: Post-Translational Modifications on UBC9 in IMR90 cells.***

(a) UBC9 is a heavily post-translationally modified protein as shown by the distribution of SUMOylation, Ubiquitylation and Acetylation sites identified on UBC9 from IP and LC-MS/MS experiments. (b) Modified sites depicted on the UBC9 crystal structure (3UIP), using the color scheme from (a).


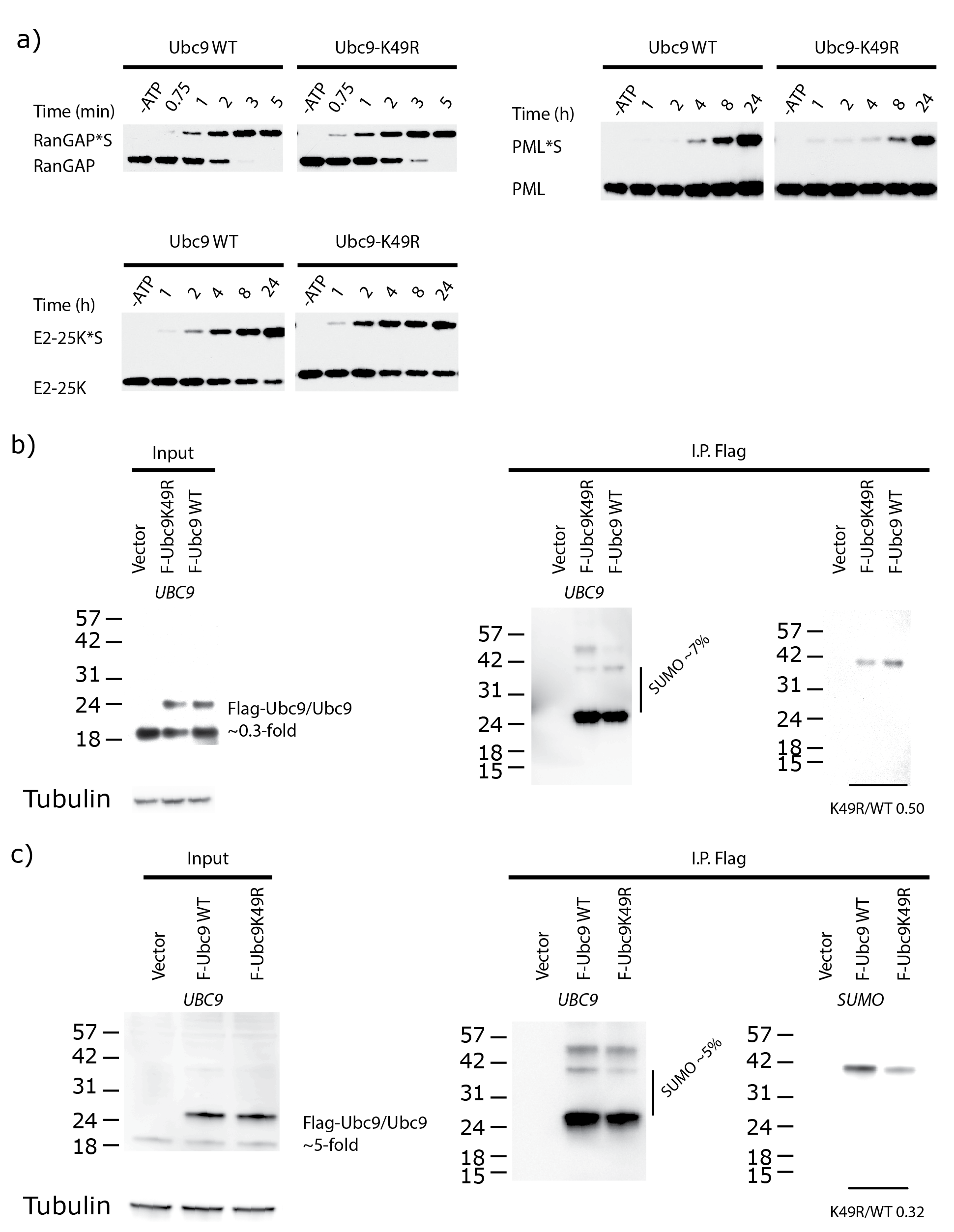


***Supplementary Figure S5:*** ***SUMOylation of UBC9 Occurs Primarily at Lys-49 and Does not Affect its Catalytic Activity.***

(a) The K49R alteration on UBC9 does not affect its catalytic activity *in vitro*. Time course SUMOylation of RanGap, of a SUMOylatable peptide of PML and of E2-25K by wild type Ubc9 (Ubc9 WT) and the K49R variant (Ubc9-K49R). (b) Flag-IP results from U2OS cells expressing SUMO3m and a control vector, Flag-Ubc9 (F-Ubc9 WT) or its K49R variant (F-Ubc9K49R) showing Lys-49 as a main SUMO acceptor site. (c) Flag-IP results from IMR90 cells expressing SUMO3m and a control vector, Flag-Ubc9 (F-Ubc9 WT) or its K49R variant (F-Ubc9K49R) showing Lys-49 as a main SUMO acceptor site.

***
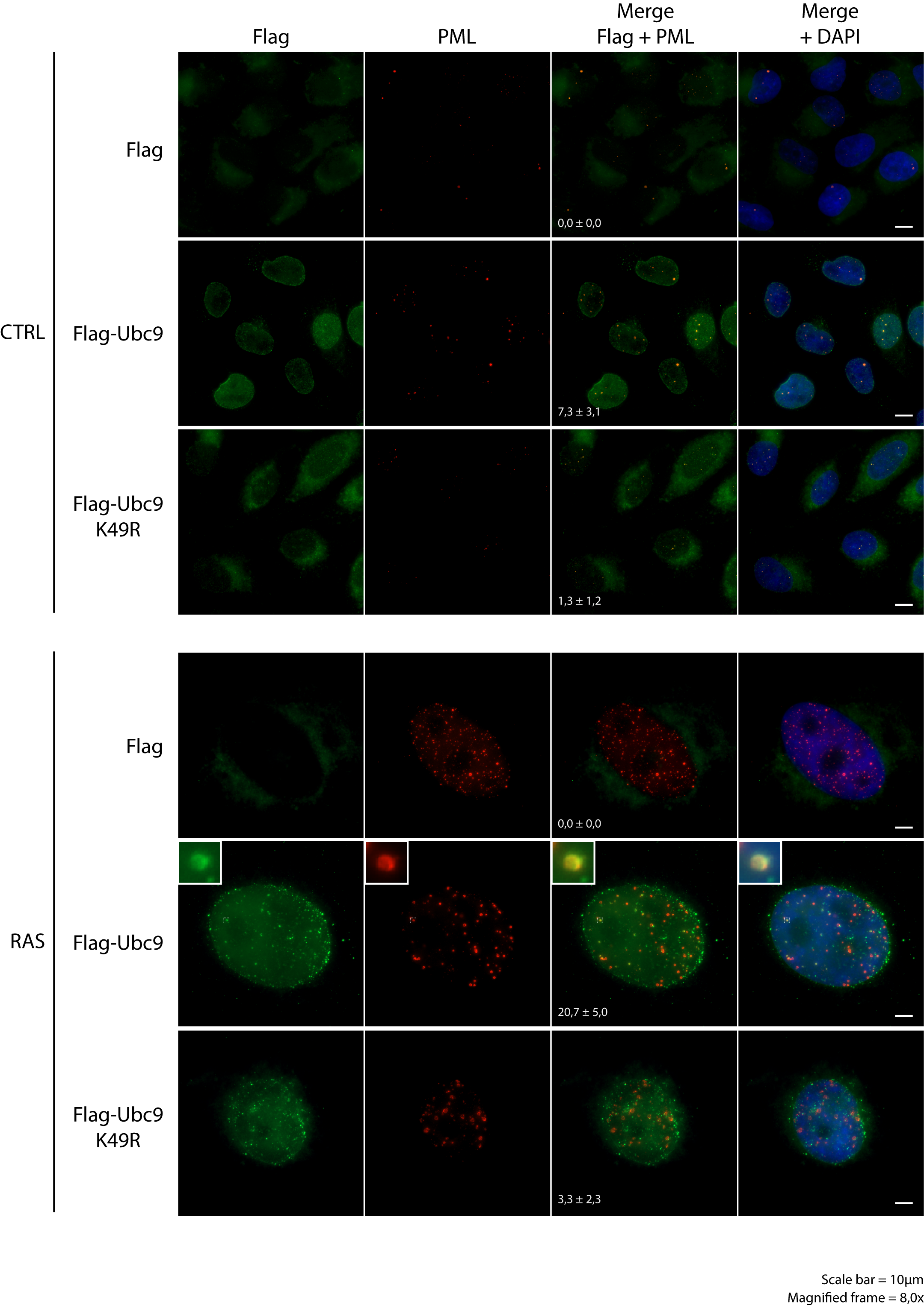
***

***Supplementary Figure S6: Immunofluorescence of F-UBC9 WT Co-localizing into PML-NBs More Readily than F-UBC9-K49R Upon RAS Mediated Senescence.***

Double immunofluorescence with anti-FLAG and anti-PML antibodies to show colocalization of F-UBC9 WT (wild type) or K49R variant in U2OS cells expressing SUMO3m plus (a) control vector or (b) H-RAS-V12 (RAS) to induce senescence. Percent of cells considered positive for Flag/PML colocalization - with at least 4 common foci - is indicated in the “Merge Flag + PML” panels. DAPI: DNA counterstain. (scale bar: 10 μm, Magnified frame = 8.0x)


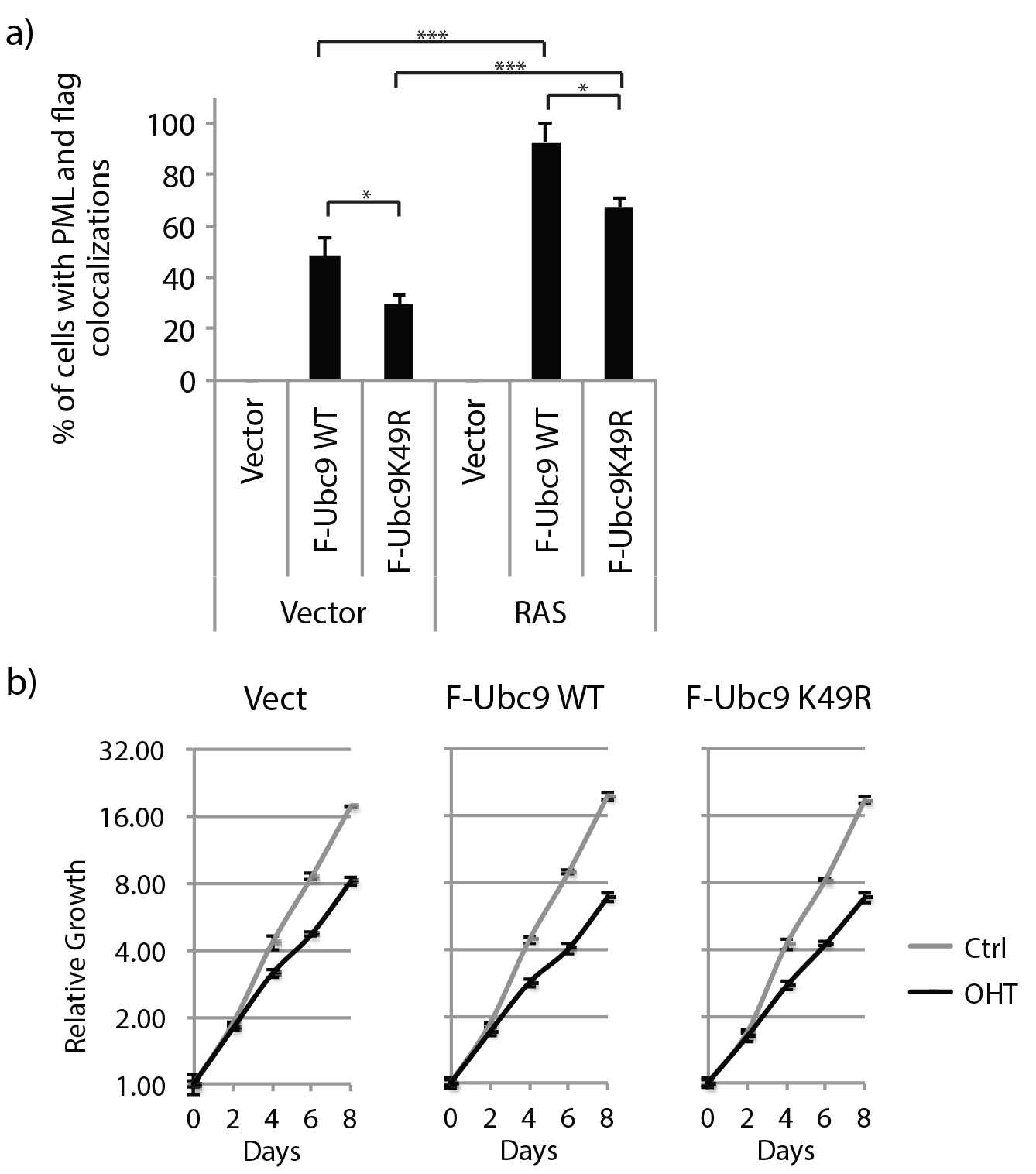


***Supplementary Figure S7: SUMOylation of UBC9 at Lys-49 Promotes its Association to PML-NBs but does not Delay Senescence in IMR90 Cells.***

(a) IMR90 cells stably expressing SUMO3m and wild type Ubc9 (F-Ubc9 WT) or its K49R variant (F-Ubc9K49R) were transduced with and empty vector (Vector) or H-RAS-G12V expressing vector (RAS). Ten days after transduction, the cells were fixed for immunofluorescence. Quantification for the colocalization of UBC9 and PML were obtained from immunofluorescence analyses using monoclonal mouse anti-Flag and rabbit anti-PML antibodies. (b) Growth curves of IMR90 cells expressing SUMO3m and an Er-H-RAS-G12V construct (fusion of the ligand-binding domain of the estrogen receptor with H-RAS-G12V to control its activity with 4-hydroxy-tamoxifen: OHT, used at 100nM) (Er-RAS) and transduced with a control vector, Flag-Ubc9 wild type (F-Ubc9 WT) or Flag-Ubc9 with the K49R mutation (F-Ubc9 K49R).

***
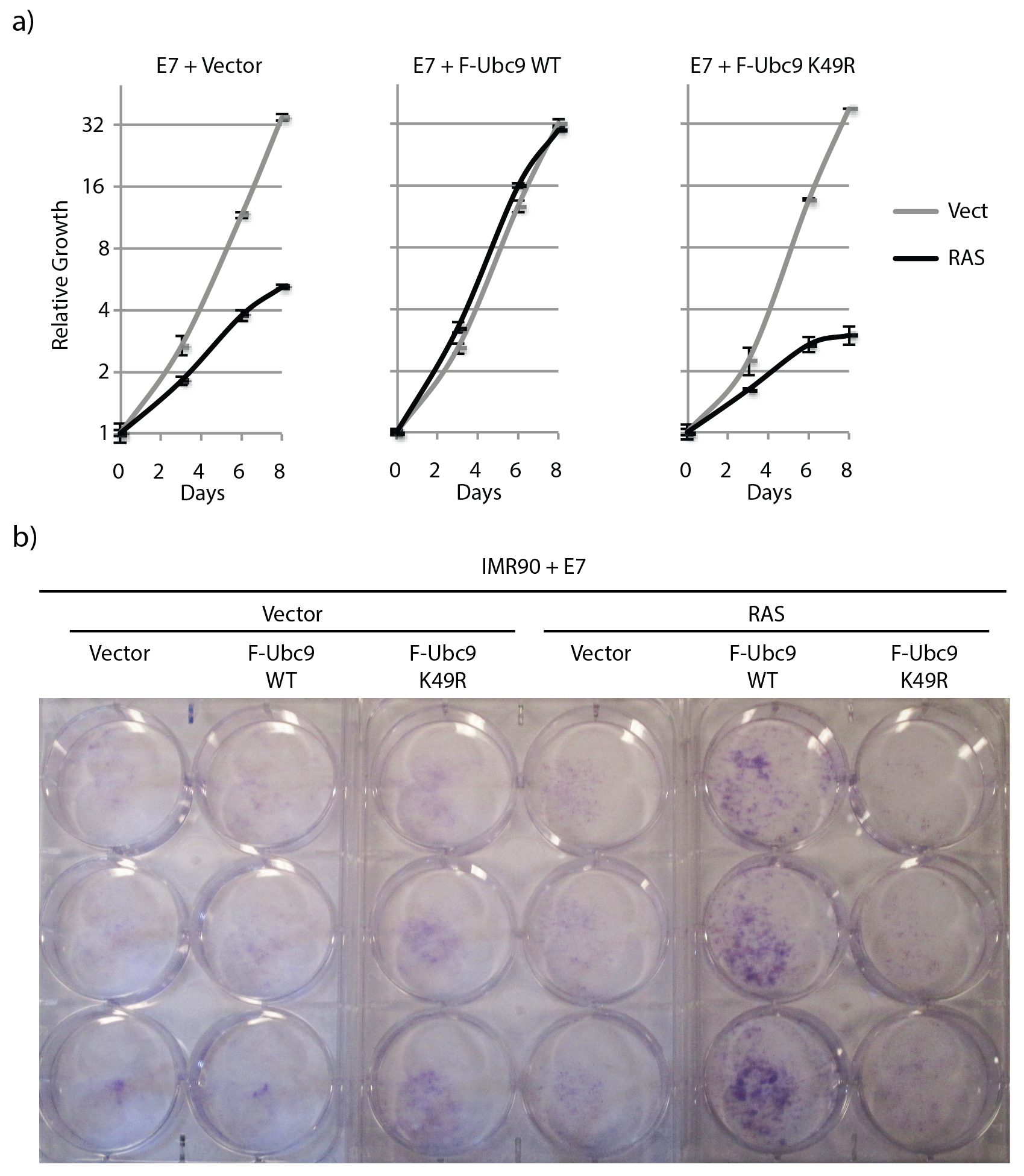
***

***Supplementary Figure S8: SUMOylation of UBC9 at Lys-49 Bypasses Senescence in IMR90 Cells when the RB/E2F pathway is Inhibited.***

(a) Growth curves of IMR90 cells stably expressing wild type Ubc9 (F-Ubc9 WT) or its K49R variant (F-Ubc9K49R) that were transduced with and empty vector (Vector) or H-RAS-G12V expressing vector (RAS) while inhibiting the RB/E2F pathway through expression of the E7 viral protein. (b) Crystal violet stained petri from which the quantification for panel a) were derived showing the staggering bypass of the senescence phenotype when IMR90 cells expressed the WT Ubc9 construct in cells with a compromised RB/E2F pathway.

***
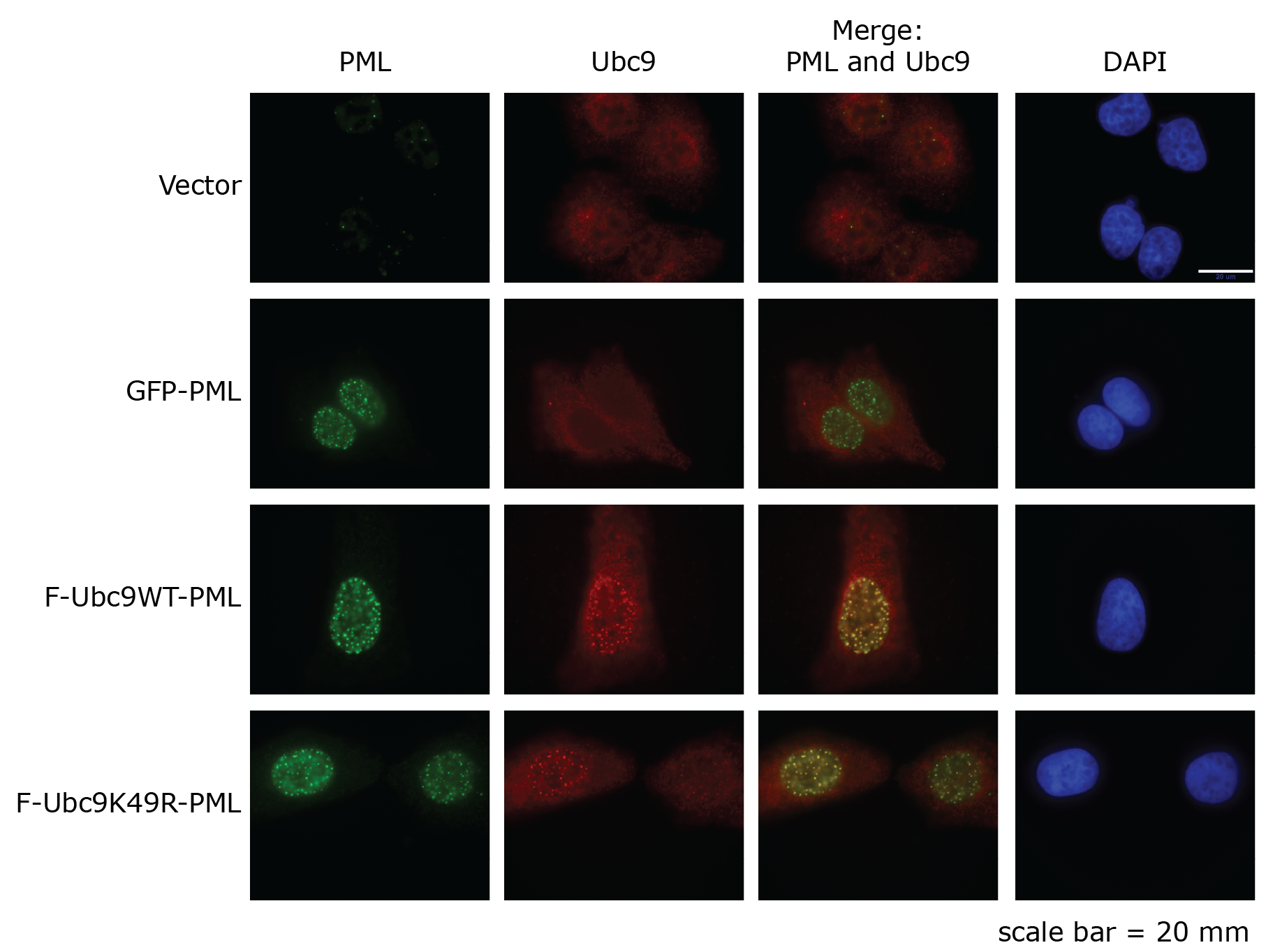
***

***Supplementary Figure S9: PML Fusion Proteins Localize to PML-NBs.***

Double immunofluorescence with anti-PML (green) and anti-UBC9 (red) antibodies to show colocalization signals for F-Ubc9 wild type (WT) or its K49R variant with the PML signal in U2OS cells expressing SUMO3m and either control vector, or one of the PML fusion proteins. DAPI: DNA counter stain. Scale bar: 20 μm.

***
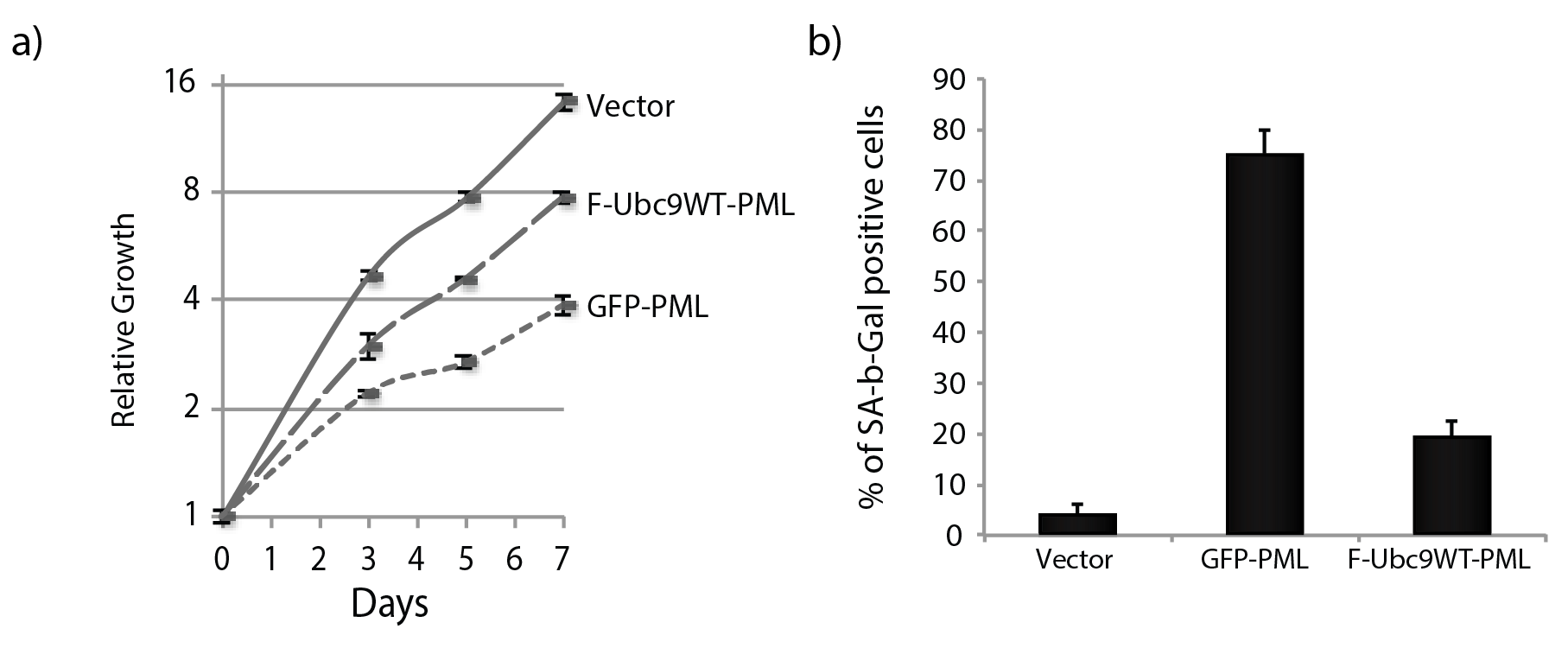
***

***Supplementary Figure S10: UBC9 Provides Anti-senescent Properties when Forced to PML-NBs in IMR90 cells.***

(a) Growth curves of IMR90 cells stably expressing SUMO3m and a control vector, GFP-PML fusion or a fusion of PML with wild type UBC9 (F-Ubc9WT-PML). (b) Senescence-associated β-galactosidase (SA-b-Gal) assay results supporting the results from panel (a), where GFP-PML induces a senescent phenotype, while expression of the F-Ubc9WT-PML construct partially alleviates this senescence.

***
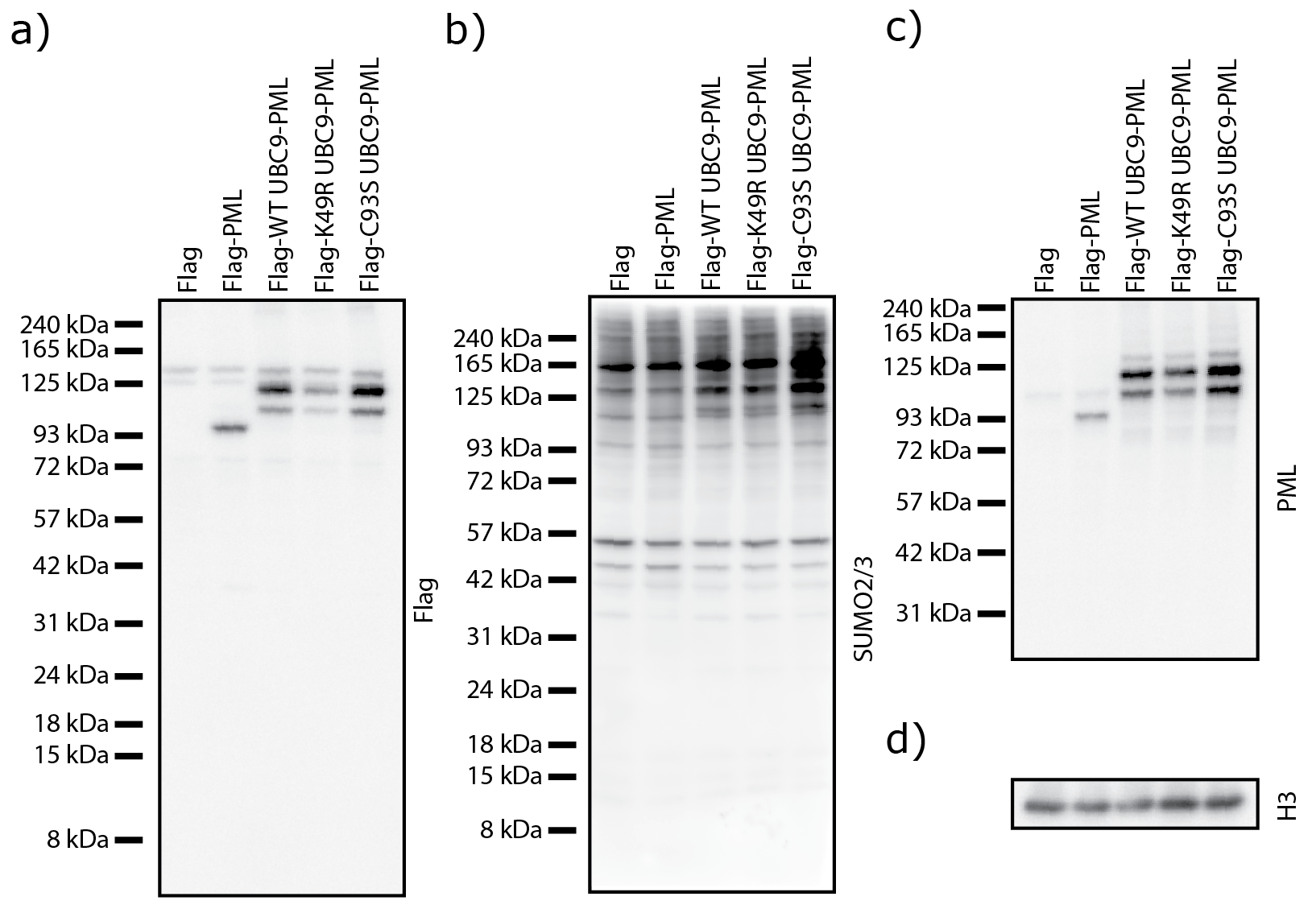
***

***Supplementary Figure S11: Ubc9-PML Fusion Protein Expression do not Increase the SUMO Proteome of the Cells.***

Western blot analysis of whole cell extracts to study the effects of the Ubc9-PML fusion proteins on the SUMO proteome. (a) Anti-Flag western blotting showing a similar level of expression of the various constructs in the U2OS cells. (b) Anti-SUMO2/3 blotting depicting the constant level of conjugated SUMO2/3 in the various cells. (c) Anti-PML blotting to validate that the results from panel a were PML in nature since PML is at the C-terminus of the fusion constructs. (d) Anti-H3 loading control for all previous panels.


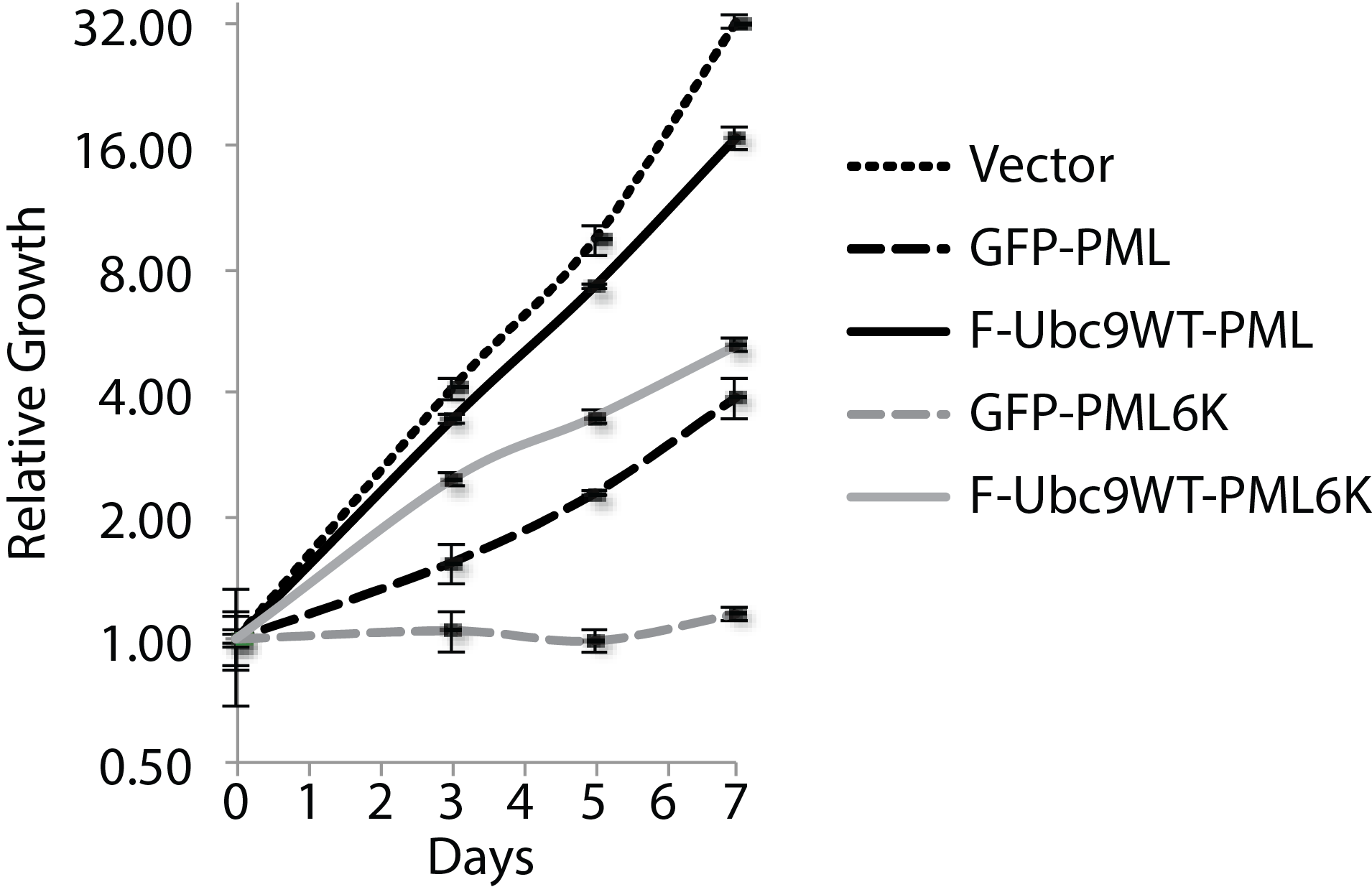


***Supplementary Figure S12: UnSUMOylatable PML induces senescence but its fusion with Ubc9 bypasses the phenotype.***

Growth curves of U2OS cells stably expressing SUMO3m and a control vector, a GFP-PML fusion, a fusion of PML with wild type UBC9 (F-Ubc9WT-PML), a GFP-PML6K fusion (6 lysine residues known to be SUMOylated are converted to arginine to abolish PML SUMOylation – K65, K160, K380, K478, K490 and K497) or a fusion of PML6K with wild type UBC9 (F-Ubc9WT-PML6K).

***
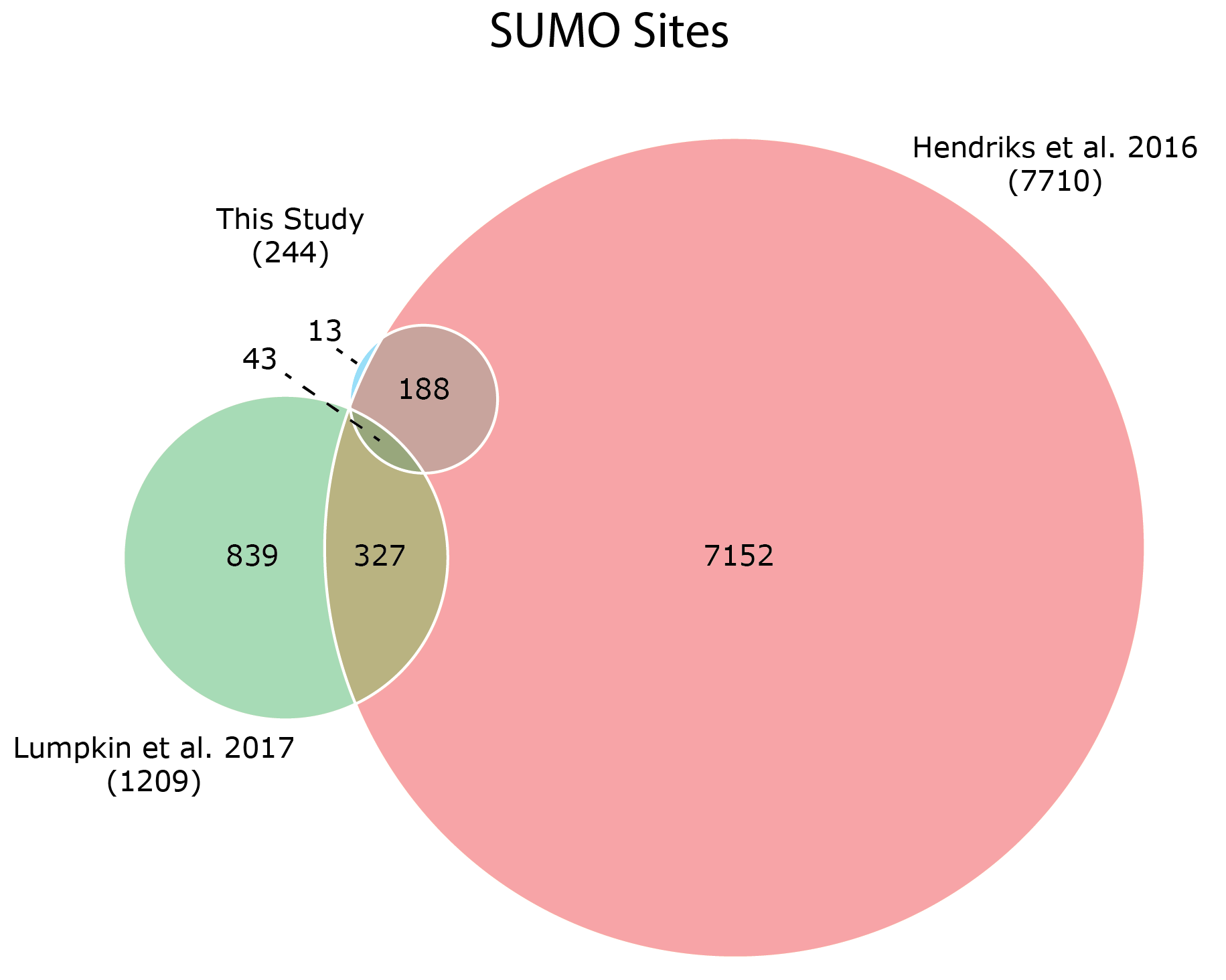
***

***Supplementary Figure S13: Overlap of SUMO Sites Identified in this Study to those Identified in Past Large Scale Proteomics Studies (Hendriks et al.) and those Identified Endogenously using the WaLP Procedure (Lumpkin et al.).***


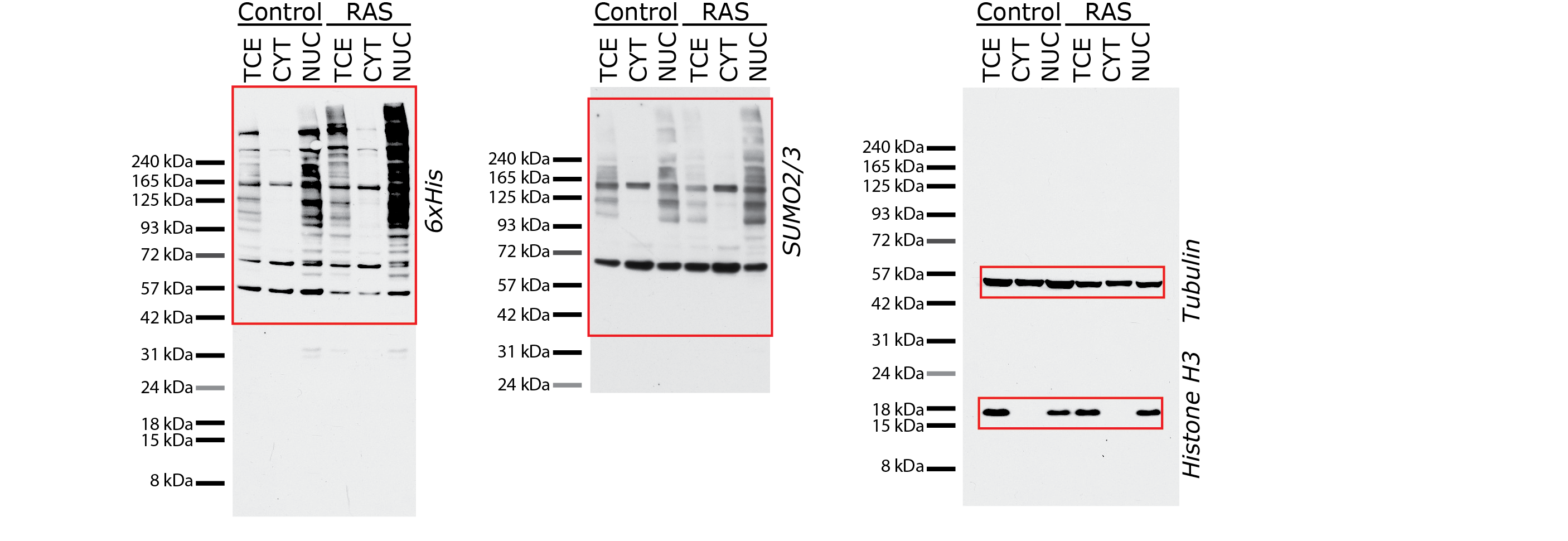


***Supplementary Figure S14: Uncropped Western blots for Figure 1.***

***
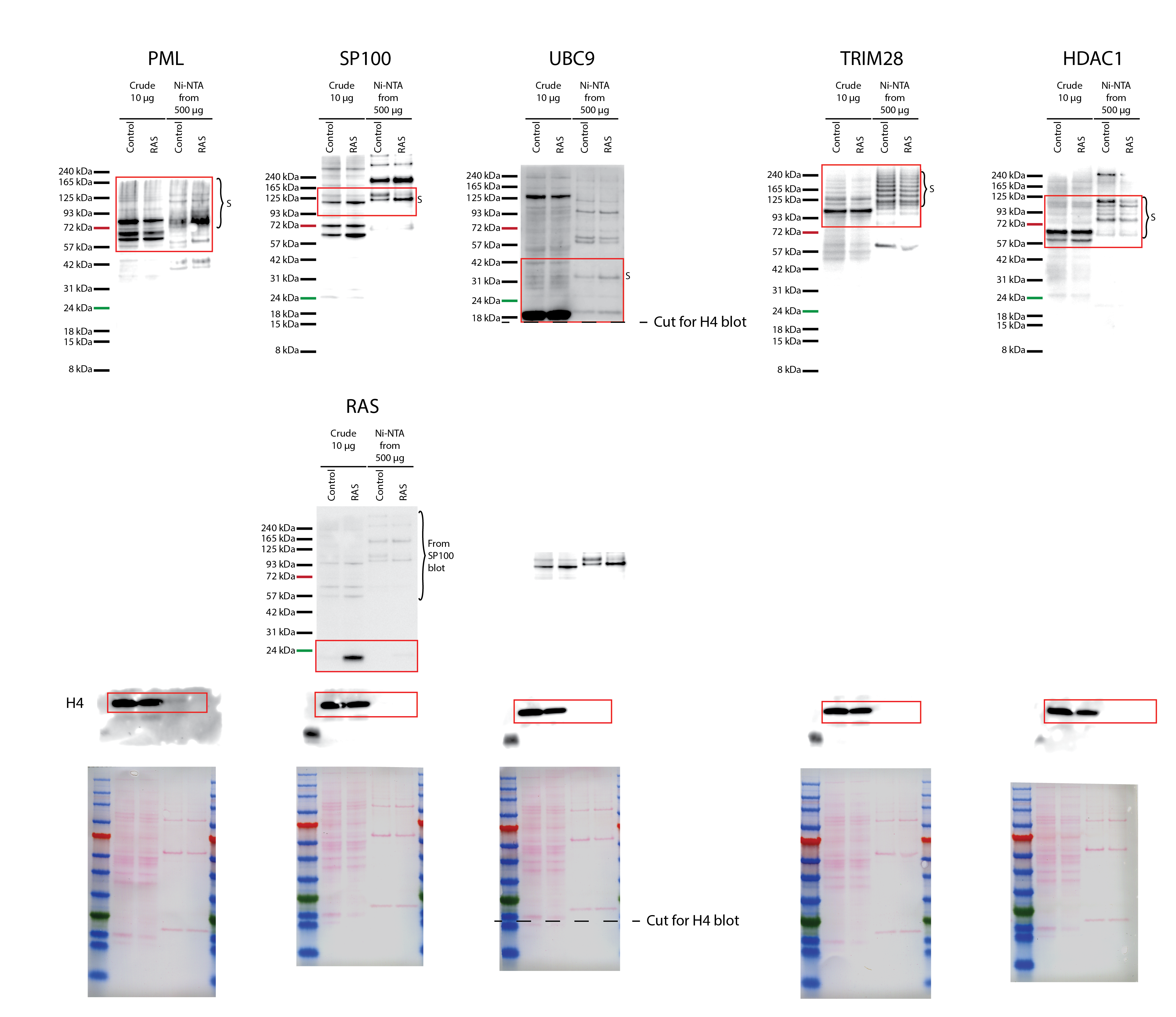
***

***Supplementary Figure S15: Uncropped Western blots for Figure 2.***

***
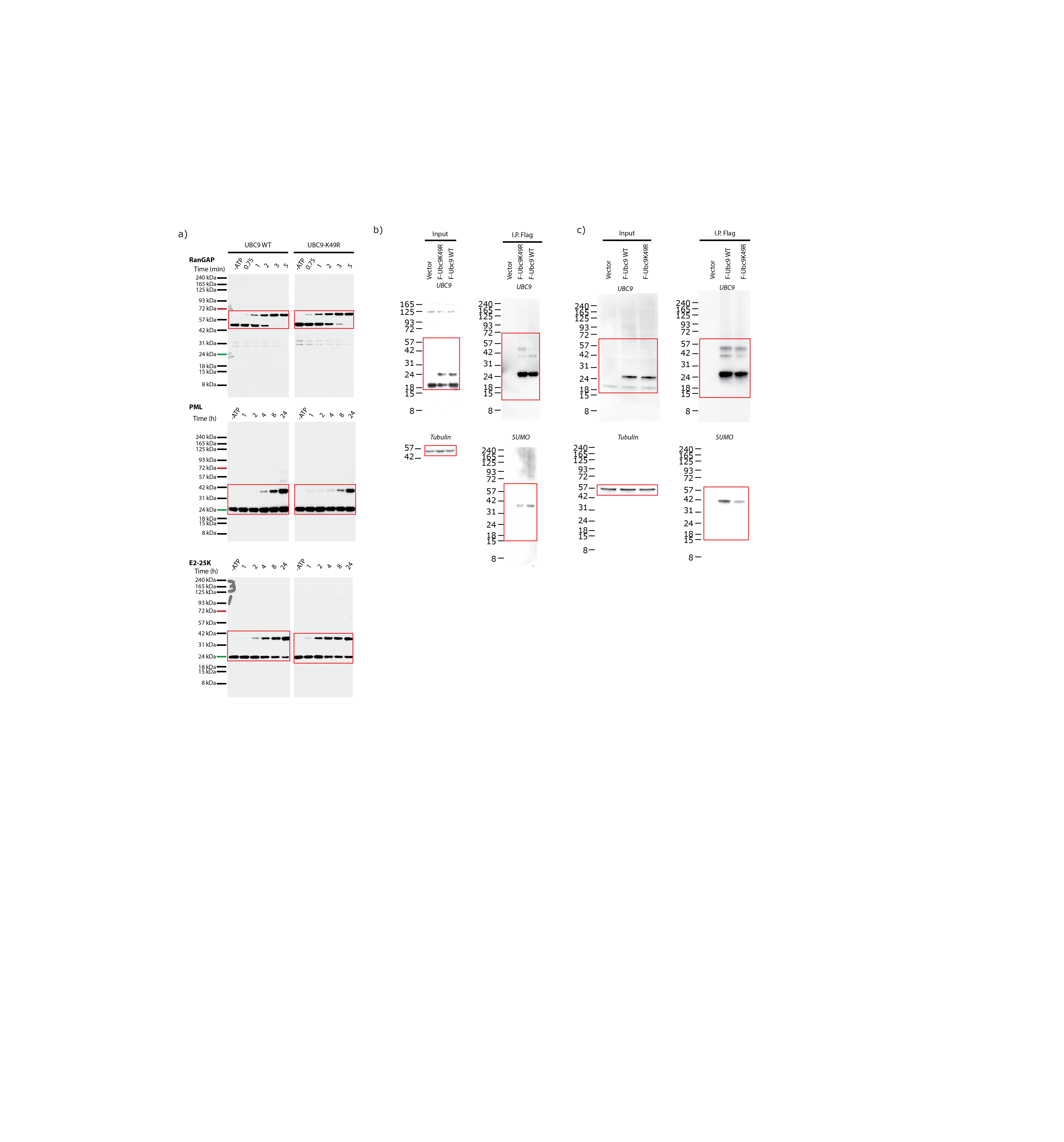
***

***Supplementary Figure S16: Uncropped Western blots for Supplementary Figure S5.***
